# Reclassification of Gall Midges (Diptera: Cecidomyiidae: Cecidomyiini) from Amaranthaceae, with Description of Ten New Species Based on an Integrative Taxonomic Study †

**DOI:** 10.3390/insects12121126

**Published:** 2021-12-16

**Authors:** Netta Dorchin, Einat Shachar, Ariel Leib Leonid Friedman, Omri Bronstein

**Affiliations:** 1 School of Zoology, The George S. Wise Faculty of Life Sciences, Tel Aviv University, Tel Aviv 6997801, Israel; einat.laor@gmail.com (E.S.); bronstein@tauex.tau.ac.il (O.B.); 2 The Steinhardt Museum of Natural History, Tel Aviv University, Tel Aviv 6997801, Israel; laibale@tauex.tau.ac.il

**Keywords:** *Asiodiplosis*, bud galls, *Halodiplosis*, host plant, Salsoloideae

## Abstract

**Simple Summary:**

Plants of the family Amarantaceae are hosts to hundreds of gall-midge taxa (Diptera: Cecidomyiidae), one of which is the genus *Halodiplosis*, which has been known so far from 99 species, almost all of which are from Central Asia. Immature stages have never been described in this genus nor have molecular data been used in its classification. In preparation for the description of 10 new species from Israel, we conducted a thorough review of the taxonomic literature on *Halodiplosis* (almost all of which is in Russian) and found that this genus should be divided into several genera based on morphological and life-history attributes. As a result, we reinstated the genus *Asiodiplosis* for most of the species in this group, all of which develop in complex bud galls. This leaves only 14 species in *Halodiplosis*, which live in galls induced by other gall-midges or develop in plant tissues without causing distinct galls. Here we describe nine *Asiodiplosis* species and one *Halodiplosis* species from Israel based on morphological and molecular data, including the first description of larvae and pupae for these genera. This work demonstrates the value of combining morphological, molecular, and life-history data to resolve the systematics of taxonomically difficult groups.

**Abstract:**

The genus *Halodiplosis* includes 99 species restricted to host-plants of the Amaranthaceae, virtually all of which are from Central Asia. The discovery of numerous undescribed species putatively belonging to this genus in Israel instigated an exhaustive review of the original descriptions of all known species in this genus. This study revealed that the generic concept of *Halodiplosis* and some of the genera synonymized under it should be redefined based on morphological and life-history attributes, such that *Halodiplosis* is limited to only 13 species developing in plant tissues without obvious gall formation or as inquilines in galls of other cecidomyiids. Revised status were proposed for *Asiodiplosis*, *Onodiplosis*, and *Desertomyia*, all species of which are gall inducers. A detailed morphological study of the Israeli species combined with data on their life history and an analysis of mitochondrial COI and 16S gene sequences revealed nine gall-inducing species belonging to *Asiodiplosis* and one inquilinous species belonging to *Halodiplosis*. All ten species (*Asiodiplosis admirabilis* n.sp., *A. bimoda* n.sp., *A. delicatula* n.sp., *A. largifica* n.sp., *A. mohicana* n.sp., *A. mucronata* n.sp., *A. paradoxa* n.sp., *A. pillosaeconspicua* n.sp., *A. stellata* n.sp., and *Halodiplosis fugax* n.sp.) are described here as new to science, including the first descriptions of larvae and pupae for these genera.

## 1. Introduction

Gall midges (Diptera: Cecidomyiidae) constitute the largest and most diverse group of gall-inducing insects and are associated with a great number of host-plant families all over the world [1,2]. Certain plant families, including Asteraceae, Fabaceae, Fagaceae, and Salicaceae, support particularly high numbers of cecidomyiid species [3]. In Central Asia and the Mediterranean Basin, certain subfamilies of Amaranthaceae (those formerly included in the Chenopodioiceae—termed “chenopods” hereafter) constitute some of the most dominant groups of host-plants for gall midges, supporting hundreds of cecidomyiid species in deserts and salt-marsh habitats [4]. Cecidomyiid taxa of at least four tribes have diversified on these plants independently [5], resulting in a great diversity of galls on all plant organs, with certain plant species hosting more than 20 gall-midge species [2,4]. One example of this radiation is the genus *Halodiplosis* Kieffer (Cecidomyiini), in which virtually all 99 described species form bud galls on chenopods [2]. Contrary to many cecidomyiid taxa on chenopods that develop in inconspicuous galls or cause no visible deformation [4], *Halodiplosis* galls are often conspicuous, complex, and at least locally common (e.g., [6,7,8,9,10]). The overwhelming majority of *Halodiplosis* species are associated with host plants of the subfamily Salsoloideae, and more than 70% of them are found on *Salsola* L. (sensu lato), *Anabasis* L., or *Haloxylon* Bunge. The type species, *H. salsolae* Kieffer, was described from Tunisia [11], and a single species, *H. sarcobati* Felt, is known from North America [12]. All remaining species are found in Central Asia (mostly Kazakhstan and Turkmenistan), which may be the center of speciation for this cecidomyiid genus.

Descriptions of new species within *Halodiplosis* relied mostly on morphological attributes of female and male terminalia, antennal flagellomeres, and galls. Larvae and pupae have never been described, molecular data have not been used, and information on the life history of many species is lacking. Some species were described only from one of the sexes (e.g., [11,13,14,15]), and many descriptions did not include illustrations of the insects (e.g., [16,17]), or were accompanied by simplistic, non-diagnostic illustrations, making it difficult to appreciate the relevant characters. More than ten genera erected for Central-Asian species (e.g., *Haloxylophaga* Marikovskij [6], *Asiodiplosis* Marikonvskij [6], and *Bojalodiplosis* Fedotova [18]) were synonymized under *Halodiplosis* by Gagné [19,20] and are listed under it in the world catalog of Cecidomyiidae [2].

In this study, we describe ten new species from chenopod host-plants in Israel based on data collected over more than 25 years. We provide descriptions of adults of both sexes, the first descriptions of larvae and pupae, and information on the galls and life histories of all species. The integrity of Israeli species is supported by a phylogenetic analysis of sequences from the mitochondrial COI and 16S genes. Based on a thorough revision of the relevant literature, we reinstate the genera *Desertomyia* Marikovskij for two species that develop on Fabaceae rather than Amaranthaceae, *Onodiplosis* Felt for the single North-American species, and *Asiodiplosis* for most of the Asian species. We also provide revised generic concepts for all genera involved, leaving 14 species in the newly delimited *Halodiplosis*.

## 2. Materials and Methods

### 2.1. Collecting and Rearing of Insects

**Figure 1:**
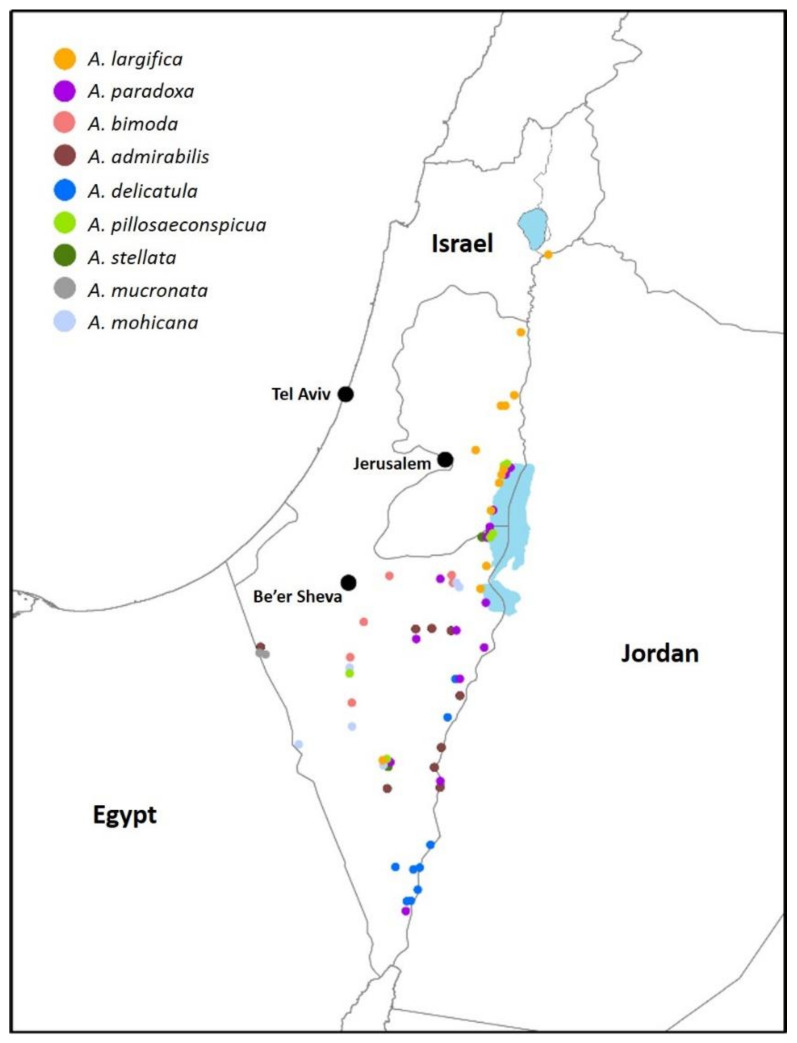
Distribution map of *Asiodiplosis* species in Israel.

Field collections of galls were conducted between 1995 and 2021 at all times of the year, in numerous sites in Israel, including the central Jordan Valley, the Judaean Desert, the Negev Desert, and the Arava Valley (Figure 1). Galls were collected into plastic bags and brought to the laboratory, where they were placed in ventilated rearing cages until adult emergence. A subset of the galls was dissected under a stereo-microscope to learn about the gall structure and to obtain larvae and pupae for the morphological analysis. Adults and immature stages were preserved in 70% ethanol for morphological analysis or in 96% ethanol for molecular analysis. Adults, larvae, and pupal exuviae were mounted on permanent microscope slides in Euparal according to the procedure outlined by Gagné [21]. Pupae were studied by scanning electron microscope (SEM)-imaging following chemical drying and gold sputtering.

### 2.2. Taxonomy

Characters of the newly described species were compared to those of the known *Halodiplosis* species based on a thorough review of the published descriptions and illustrations. The actual type specimens have not been examined. Images of adult morphological characters were taken with a Leica DFC 495 camera (Leica Microsystems, Wetzlar, Germany) connected to a Leica M205-C stereo microscope (Leica Microsystems, Wetzlar, Germany), drawn with a drawing tube connected to a Leica DM1000 compound microscope (Leica Microsystems, Wetzlar, Germany), or taken with a JEOL JCM6000 benchtop scanning-electron microscope (JEOL, Tokyo, Japan). Images of pupae were taken with a Thermo Scientific Phenom XL (Thermo Fisher Scientific Inc., Waltham, MA USA) or a JEOL JCM6000 benchtop scanning-electron microscope. Ovipositor length was measured from the posterior margin of the 8th abdominal tergite to the end of the fused cerci and expressed relative to the length of the 8th abdominal tergite. Terminology for adult morphology follows McAlpine [22]; for wing venation, Cumming and Wood [23]; and for larval morphology, Gagné [3].

Holotypes and most paratypes are deposited at the Steinhardt Museum of Natural History, Tel Aviv University, Israel (SMNHTAU). Some paratypes are deposited at the Zoologisches Forschunginstitut und Museum Alexander Koenig, Bonn, Germany (ZFMK) as indicated under the material examined sections of the descriptions below.

Plant names used in this study are based on information in the Flora of Israel Online platform (https://flora.org.il/en/en/) (accessed on 10 December 2021) and recognizing nomenclatorial changes in Akhani et al. [24].

### 2.3. Molecular Methods

The dataset analyzed here included 62 samples of putative ingroup taxa within *Asiodiplosis*, and with *Contarinia loti* De Geer as an outgroup. The single *Halodiplosis* species from Israel is rare and was unavailable for this analysis. Individuals included in the analysis were meant to represent the distribution range and phenology of each putative species as much as possible. The molecular analysis was used to corroborate the morphological data and verify species’ identities rather than infer phylogenetic relationships among them. Collecting data for the samples and the respective Genbank accession numbers are given in Table 1.

Genomic DNA was extracted from whole adult individuals, larvae, or pupae using the Genaid Genomic DNA Mini Kit (Genaid, Taipei, Taiwan). A 658 bp fragment from the 5′ end of the mitochondrial COI gene was amplified using the primers LCO1490 and HCO2198 [25] and a ~560 bp fragment from the 5′ end of the mitochondrial 16S gene using the primers 16S1472-JJ and 16S-ar-JJ [26]. PCR conditions for both gene fragments consisted of 10 min initial denaturation at 95 °C followed by 35 cycles of 30 s denaturation at 95 °C, 1 min annealing at 50 °C, 1 min extension at 72 °C, and final extension at 72 °C for 4 min. PCR reactions were performed on a 2720 Thermal Cycler (Applied Biosystems, Foster City, CA, USA). PCR products were verified on a 1% TAE agarose gel and successful amplifications were purified using an EXO-SAP enzymatic cleanup (Thermo Scientific, Vilnius, Lithuania). Sequencing was carried out using the BigDye terminator v1.1 Cycle Sequencing Kit (Applied Biosystems, Foster City, CA, USA) on an ABI PRISM 3730xl DNA analyzer and Sequencing Analysis Software v5.2 at Hy Laboratories, Rehovot, Israel.

### 2.4. Phylogenetic Analysis

Forward and reverse sequences of each locus were assembled, inspected, and edited using SeqTrace [27]. Consensus sequences were edited using AliView v.1.18 [28] and aligned using MAFFT v.7 alignment server (http://mafft.cbrc.jp/alignment/server/) (accessed on 19 September 2021), employing the E-INS-i algorithm. Ambiguous positions were identified using TrimAl v.1.4 [29] and GUIDANCE2 [30], followed by manual inspection and removal. The final aligned sequence lengths were COI: 564 bp (*n* = 67) and 16S: 352 bp (*n* = 46). We concatenated all loci into a single dataset that was partitioned in downstream analyses by locus and codon position (for COI).

Phylogenetic analyses were conducted using both maximum-likelihood (ML) and Bayesian-inference (BI) approaches. Preliminary ML analyses were conducted with each locus separately using the default parameters on IQtree2 [31] to assess concordance and identify potential errors. A heuristic search under the Bayesian information criterion (BIC) [32], as implemented in PartitionFinder2 [33], was employed to determine the optimal partitioning schemes and models of molecular evolution for the phylogenetic analyses.

ML analyses on the concatenated dataset were performed independently with IQtree2 and raxmlGUI 2.0 [34]. IQtree2 analyses used ModelFinder [35] to select the best models for each partition, identifying the TIM + F + G4 as the best-fit model across all partitions. We evaluated branch support with ultrafast bootstrap (UFBoot, 1000 reps.) as well as standard bootstraps (BS, 1000 reps.), Shimodaira–Hasegawa approximate likelihood ratio tests (SH-aLRT, 1000 reps.), and approximate Bayes (aBayes) tests. An additional ML analysis performed with raxmlGUI 2.0 applied the settings “ML + thorough bootstrap,” 100 runs, 1000 replicates, using GTR + G as the best-fit model for all partitions as inferred from PartitionFinder2.

Bayesian analysis was carried out using MrBayes 3.2.6 [36]. We conducted two independent runs of three “heated” and one “cold” chain for 10 million generations and sampled parameters and trees every 100 generations. The runs were inspected with Tracer 1.7.1 [37] to assess the behavior of the runs, and convergence was assessed using the RWTY package [38] implemented in R 4.0.5 [39]. In a conservative approach, the first 25% of trees were discarded as burn-ins, and a 50% majority-rule consensus tree was calculated from the remaining trees. Bayesian posterior probabilities (PP) were obtained from the 50% majority-rule consensus of the trees sampled during the stationary phase.

Based on the COI dataset, uncorrected mean p-distances and Kimura two-parameter (K2P; [40]) genetic distances were calculated within and between species/clades using MEGA v.7.0.26 [41].

## 3. Results

### 3.1. Systematics of Halodiplosis

A review of the descriptions of all 99 described species listed under *Halodiplosis* in the world catalog of Cecidomyiidae [2] revealed that the genus groups several distinct entities that merit separate generic status. As currently circumscribed, the majority of species in *Halodiplosis*, including 9 of the 10 Israeli species described here, are characterized by binodal and bifilar antennal flagellomeres in the males (two circumfilar loops on each flagellomere) and a greatly reduced, one-segmented palpus. By contrast, 14 species, including the type species *Halodiplosis salsolae* and one Israeli species described here, have trifilar male flagellomeres (three circumfilar loops on each flagellomere), which is a situation that is always correlated with a four- or three-segmented palpus. The single described North-American species, *Halodiplosis sarcobati*, has trifilar male flagellomeres but one-segmented palpus—a third combination of characters. This latter combination is also found in *Halodiplosis dissimmetrica* Marikovskij, the only species in this genus associated with a host plant from the Fabaceae rather than the Amaranthaceae. Given these findings, we reinstate here the genera *Desertomyia* for the species from Fabaceae, *Onodiplosis* for the North-American species, and *Asiodiplosis* for the majority of the Asian species, leaving 14 species in *Halodiplosis* as detailed below and listed in Appendix A.

#### 3.1.1. *Desertomyia* Marikovskij, 1975

##### Type Species: *Desertomyia dissimmetrica*

Synonym: *Halodiplosis* Kieffer

This genus was erected for *D. dissimmetrica* Marikovskij from *Halimodendron halodendron* (Pall.) Voss (Fabaceae) based on ambiguous characters of the ovipositor [42], and was later joined by *D. caraganae* Fedotova (1990b [43]) from another fabaceous host plant, *Caragana frutex* (L.) K.Koch. Gagné [20] placed *D. dissimmetrica* in *Halodiplosis* and *D. caraganae* in *Contarinia*, but these species do not seem to fit in either of these genera, hence *Desertomyia* is reinstated for them here. *Desertomyia* is characterized by trifilar male flagellomeres, one-segmented palpus, glabrous ovipositor with clearly separate cerci, and a narrow, tapered male hypoproct that lacks strong apical setae. Furthermore, the association of both species with host plants of the Fabaceae rather than chenopods (Amaranthaceae), the nature of their galls, and their life-history attributes speak for their separate status. Most notably, the larvae of *D. dissimmetrica* develop in leaf galls gregariously, a situation that is never seen among species from chenopod hosts, and larvae of both *Desertomyia* species leave their galls to pupate in the soil [43,44].

#### 3.1.2. *Onodiplosis* Felt, 1916

##### Type Species: *Onodiplosis sarcobati*

Synonym: *Halodiplosis* Kieffer, 1912

This genus is reinstated here for the single described North-American species based on the combination of trifilar male flagellomeres, one-segmented palpus, and the distal part of the ovipositor covered by very long hair-like setae. It develops in bud galls on *Sarcobatus vermiculatus* (Hook.) Torr. [3], which was removed from the Chenopodiaceae and placed in its own family, Sarcobataceae [45]. The original description was based on two females only, but males of this and several undescribed species in the Smithsonian National Museum of Natural History are mentioned by Gagné [3,19]. Whether this genus is related to the Asian cecidomyiid species from chenopods or evolved convergently for bud galling on halophytic plants requires further study.

#### 3.1.3. *Halodiplosis* Kieffer, 1912

##### Type Species: *Halodiplosis salsolae*

Synonyms: *Halocnemomyia* Marikovskij, 1965; *Micrasiodiplosis* Mamaeva, 1980; *Bojalodiplosis* Fedotova, 1985.

The genus as delimited here is characterized by binodal and trifilar male flagellomeres, with distinct necks connecting the two nodes, three to four segmented palpus (when four segments, the first segment sometimes not clearly separated from the head, thus resembling a palpiger), empodium much shorter than the bend in the claw, and ovipositor with prominent cerci and relatively short, sparse setae, not longer than the ovipositor height. Pupae have not been described to date, and characters of larvae other than their dull or bright orange color are known only for the Israeli species described below. The larva is elongate, with strongly spiculose integument and well-developed, bidentate spatula. Sternal and ventral papillae are asetose, lateral papillae with very short setae, pleural and dorsal papillae bear long setae, and terminal segment with pair of large, coniform, conspicuously pigmented papillae and two pairs of setiform papillae.

Interestingly, it appears that all species included here either live in plant tissues without causing distinct galls or develop as inquilines in galls of other species on chenopods, and most leave the plant to pupate in the soil [18,45,46]. This is in stark contrast to *Asiodiplosis* species (as delimited here), all of which develop in complex bud galls and pupate inside their galls. The two supposed exceptions for this rule, *H. anabasidis* (Fedotova) and *H. salsolae*, were described as bud gallers, but we regard these records as dubious. In both cases, the specimens used for the descriptions had been received from someone other than the describer and may have developed in the galls as inquilines. The larvae of *H. anabasidis* were reported to pupate in the soil [47], which supports this assumption.

Among the 14 species included here (Appendix A), the type species from Tunisia and the newly described species from Israel are the only ones originally described under *Halodiplosis*. The 12 remaining species from Central Asia were originally described under different genera and placed in *Halodiplosis* by Gagné [20].

#### 3.1.4. *Asiodiplosis* Marikovskij, 1955b

##### Type Species: *Asiodiplosis noxia*

Synonyms: *Halodiplosis* Kieffer, 1912; *Haloxylophaga* Marikovskij, 1955b; *Tyloceramyia* Marikovskij, 1956; *Monarthropselaphus* Marikonvskij, 1957.

With the nine new species described here, this genus includes 84 species, all from bud galls on chenopod hosts in Asia (Appendix A). The male flagellomeres are binodal and bifilar, the greatly reduced palpi are one-segmented, and the ovipositor is either densely covered by very long hair-like setae or by sparse, shorter setae, and ends with minute, closely appressed cerci. In most *Asiodiplosis* species, the nodes of male flagellomeres are separated by distinct, long necks, but the nine species originally described under *Haloxylophaga* (see Appendix A) have somewhat effeminate male flagellomeres, in which the nodes are not separated by distinct necks. Intermediate forms between these two conditions occur, which favor retaining *Haloxylophaga* as part of *Asiodiplosis*. The first two antennal flagellomeres in both sexes are conspicuously longer than subsequent flagellomeres and are usually partially fused. Similarly, the two apical flagellomeres in both sexes are often fused to form a single long unit with more than two circumfilar whorls. The structure of the male hypoproct, typically with a group of strong apical setae, often provides good diagnostic characters for species in this genus. Characters of larvae and pupae other than their yellow, orange, or pinkish color are known only for the Israeli species described below. Third-instar larvae are stout, almost spherical, with protruding, sclerotized spiracles. Some species have a distinct unidentate spatula, whereas others have a rudimentary spatula or lack one altogether. Sternal papillae are asetose and lateral papillae vary in number and setation. Terminal papillae are greatly reduced and barely visible. All species develop in complex bud galls, and pupation is always inside the gall. Pupae are bulky, lack antennal and facial horns, or antennal bases bear small to minute tapered projections. Cephalic setae and prothoracic spiracle are short. Abdominal segments are covered by tiny spicules.

### 3.2. Taxonomic Descriptions

Characters that are similar among species are not repeated after the description of the first species. The order of descriptions is from the most common and widespread to the less common species. Distribution records are given for Israel, but all species probably occur in Jordan and Egypt, and possibly in other countries in the region. Main morphological characters for the species described here are given in Table 2.

#### 3.2.1. *Asiodiplosis largifica* Dorchin, New Species

**Host plants**: *Caroxylon vermiculatum* (L.) Akhani & Roalson, *C. incanescens* (C.A.Mey.) Akhani & Roalson.

**Figure 2:**
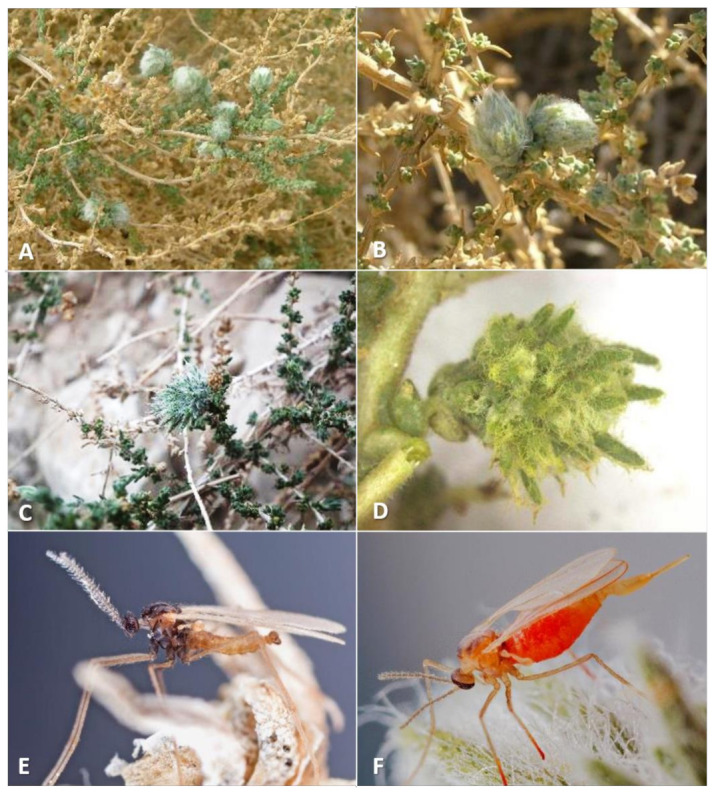
*Asiodiplosis largifica*. (**A**–**C**). Galls on *Caroxylon vermiculatum*; (**D**). Gall on *Caroxylon incanescens*; (**E**). Male; (**F**). Female.

**Gall and biology**: This is one of the most-common and widespread *Asiodiplosis* species in Israel. It develops in very-common and conspicuous bud galls on *C. vermiculatum* (Figure 2A–C)—sometimes hundreds on the same plant—and is much-less-common and conspicuous on *C. incancescens* (Figure 2D). Most galls develop in apical buds, but they are also common in lateral buds. Galls on *C. vermiculatum* vary considerably in size and shape, with some much hairier and more compact than others. Large galls can be 2 cm long and 1–1.5 cm in diameter and are less hairy, whereas smaller galls are about 6 mm long and 4 mm wide and usually contain a mass of white, wooly hairs at their center. Smaller galls are often clumped together in groups of three to five individual units. Each gall is composed of a group of soft, tapered leaves that widen at their bases, accompanied by many long, white hairs, and contains a single central chamber at its base. The side of the leaves facing the center of the gall is bright green, whereas their other side is covered by short, white hairs. The large, almost-spherical larvae and pupae fill the larval chamber almost completely. Old, empty galls remain on the plants for several months. Galls occasionally contain an unidentified inquilinous moth larva that feeds at the center of the gall inside a loose silky cocoon. On *C. incanescens*, the galls are less common and much smaller—about 5 mm in diameter, and composed of short, soft leaves that form a small rosette (Figure 2D). Empty galls remain on the plant for several months.

This species completes several generations a year. Adults were reared from *Caroxylon vermiculatum* from February to August, with a peak in early spring (February–March), and from *C. incanescens* in June to early September. This is the only Israeli species that has more than one host plant. The perennial shrub *C. vermiculatum* is apparently the primary host, whereas the annual *C. incanescens* is used by the midges in summer, when it is at its peak growth phase. Overwintering is probably in *C. vermiculatum* buds as first-instar larvae.

**Adult description**: General color: Male head and thorax grey, abdomen brownish (Figure 2E); female dull to bright orange (Figure 2F).

**Figure 3:**
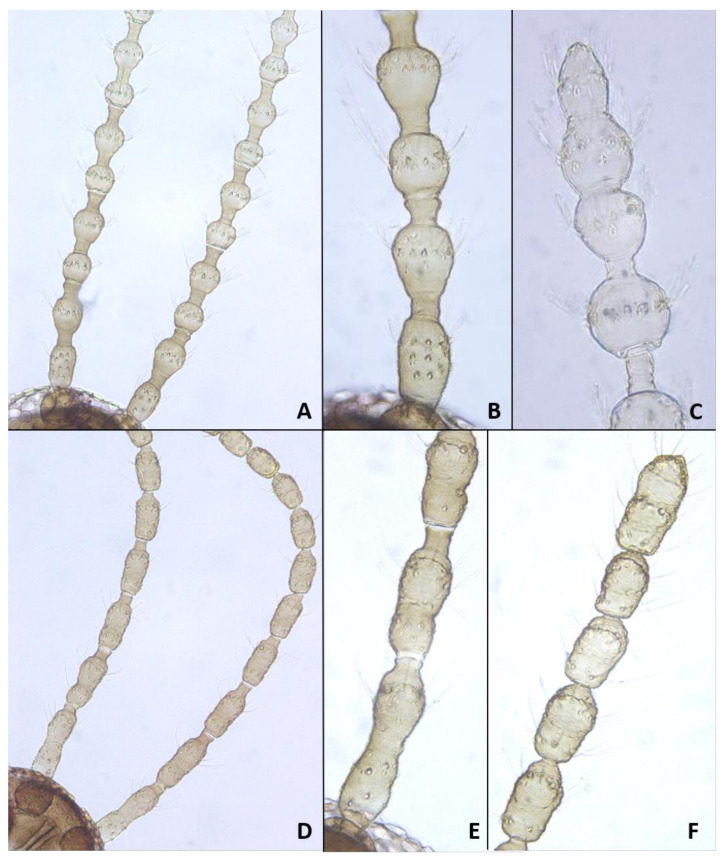
*Asiodiplosis largifica*. (**A**). Male antennae; (**B**). Male basal flagellomeres; (**C**). Male apical flagellomeres; (**D**). Female antennae; (**E**). Female basal flagellomeres. (**F**). Female apical flagellomeres.

**Figure 4:**
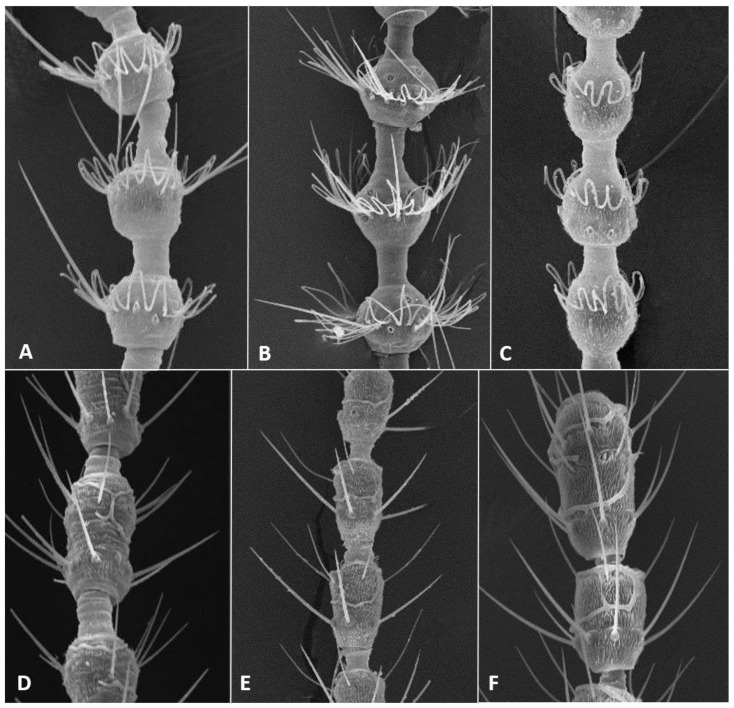
SEM images of *Asiodiplosis* flagellomeres, showing circumfila. (**A**). *A. largifica*, male mid flagellomere; (**B**). *A. delicatula*, male mid flagellomere; (**C**). *A. pillosaeconspicua*, male mid flagellomere; (**D**). *A. largifica*, female mid flagellomeres; (**E**). *A. paradoxa*, female mid flagellomeres; (**F**). *A. bimoda*, female apical flagellomeres.

**Figure 5:**
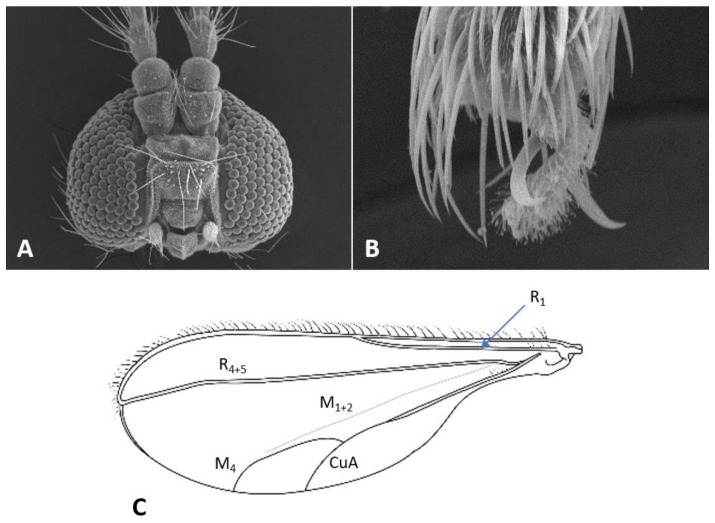
*Asiodiplosis largifica*. (**A**). Head; (**B**). Acropod, showing claws and empodium; (**C**). Wing.

*Head*: Eye facets round. Antennal flagellomeres 11–12 in both sexes; when 11, apical flagellomere composed of two fused units and is variably shaped. Male flagellomeres each composed of two nodes separated by short neck and ending with distal neck (Figure 3A) except in apical flagellomere; necks successively longer along proximal half of antenna; same length along distal half. Each node with one circumfilar whorl subtended by row of strong setae and evenly setulose (Figure 4A). Circumfilar loops about half length of node (Figure 4A). Nodes of first flagellomere cylindrical rather than spherical and not distinctly separated as nodes of subsequent flagellomeres (Figure 3B); strong setae on proximal node of first flagellomere not forming row as on subsequent flagellomeres. Apical flagellomere without distal neck, occasionally with short narrow projection, often composed of two merged units comprising three to four nodes not separated by necks (Figure 3C). Female flagellomeres cylindrical and successively shorter along antennae (Figure 3D); first five or six with distinct constriction in mid part and distinct neck, the remaining without constriction and necks. First flagellomere longer than second, sometimes partially fused with it (Figure 3E). When 11 flagellomeres (the usual case), apical flagellomere 1.5–2 times longer than preceding, appears to consist of two merged units, sometimes with small apical projection (Figure 3F). Each flagellomere with two whorls of simple, appressed circumfilla with longitudinal connections, two whorls of long setae, and otherwise evenly setulose (Figure 4D). Frontoclypeal membrane on each side with 4–8 long setae in female, 5–14 in male. Palpus 1-segmented, 1–2 times as long as wide, spherical to slightly cylindrical or wider distally than proximally, occasionally tapered, completely setulose, bearing several long setae (Figure 5A).

*Thorax*: Wing (Figure 5C) transparent, sparsely and evenly covered by fine microtrichia. R_1_ reaches C near wing mid-length, R_4+5_ straight, slightly curved before reaching C beyond wing apex; M_1+2_ present as fold; M_4_ weak, forming a fork with CuA; CuA thick along straight proximal half, weak along distal curved half. C with long hair-like setae to slightly beyond meeting point with R_4+5_; bases of R_1_, R_4+5_, and CuA with few long hair-like setae. Wing length 1.50–2.26 mm in female (*n* = 36), 1.76–2.69 mm in male (*n* = 42). Legs densely setose; claw untoothed, evenly curved, empodium extending beyond bend in claw (Figure 5B); pulvilli about third length of claw.

**Figure 6:**
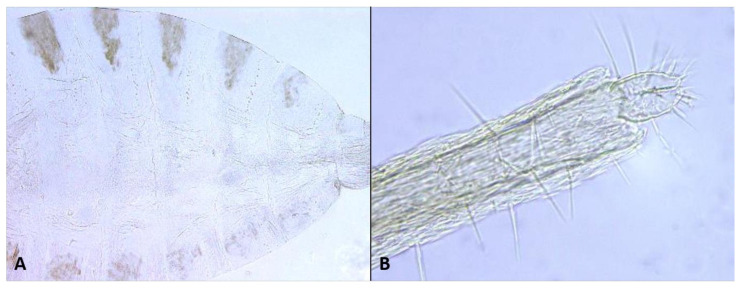
*Asiodiplosis largifica*. (**A**). Female abdomen; (**B**). Tip of ovipositor, showing closely appressed cerci.

**Figure 7:**
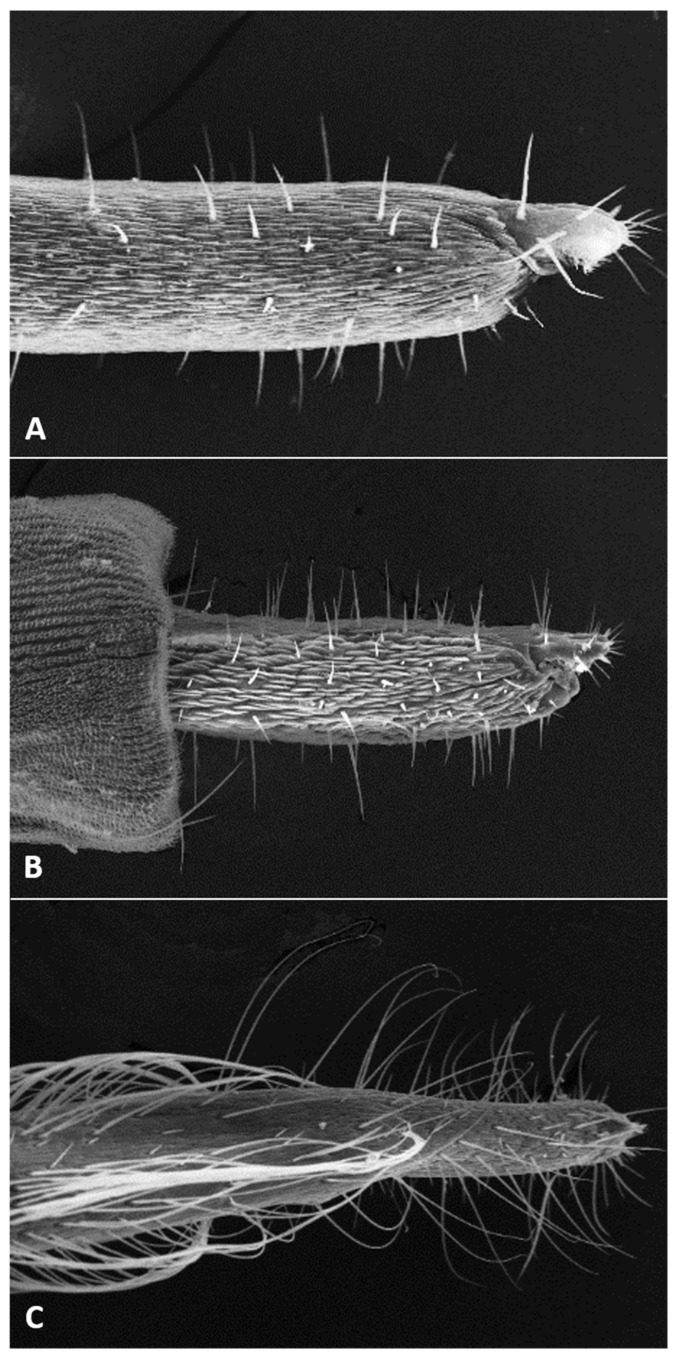
SEM images of *Asiodiplosis* ovipositors. (**A**). *A. largifica*; (**B**) *A. paradoxa*; (**C**). *A. delicatula*.

*Female abdomen* (Figure 6A): subspherical. Sclerites usually weakly pigmented. Tergites 1–7 with anterior pair of sensory setae and 1–2 posterior rows of long setae; tergite 8 with pair of sensory setae the only vestiture. Sternites 2–7 without discernible sensory setae; if pigmented, each with two separate patches of weak pigmentation and 1–2 posterior rows of long setae. Sternite eight not differentiated from surrounding tissue. Ovipositor 13.2–21.2 times as long as tergite 8 (*n* = 31); segment 8 grooved by longitudinal lines of setulae; segment 9 covered by stacked ridges, with numerous setae about 0.3 times as long as segment height (Figure 6B and Figure 7A). Cerci minute, closely appressed together to form single unit with visible seam (Figure 6B), with several setae much longer than setae on segment 9. Hypoproct about half as long as cerci.

**Figure 8:**
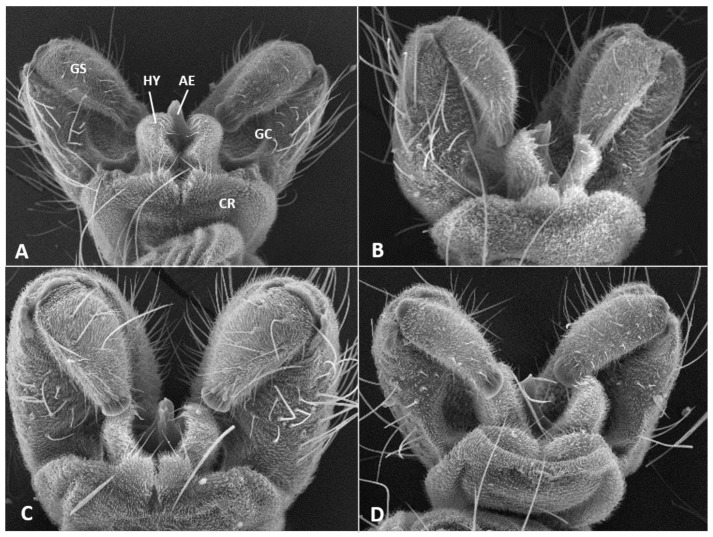
*Asiodiplosis* male terminalia, dorsal. (**A**). *A. largifica*; (**B**). *A. paradoxa*; (**C**). *A. bimoda*; (**D**). *A. pillosaeconspicua*. AE = aedeagus, CR = cerci, GC = gonocoxite, GS = gonostylus, HY = hypoproct.

**Figure 9:**
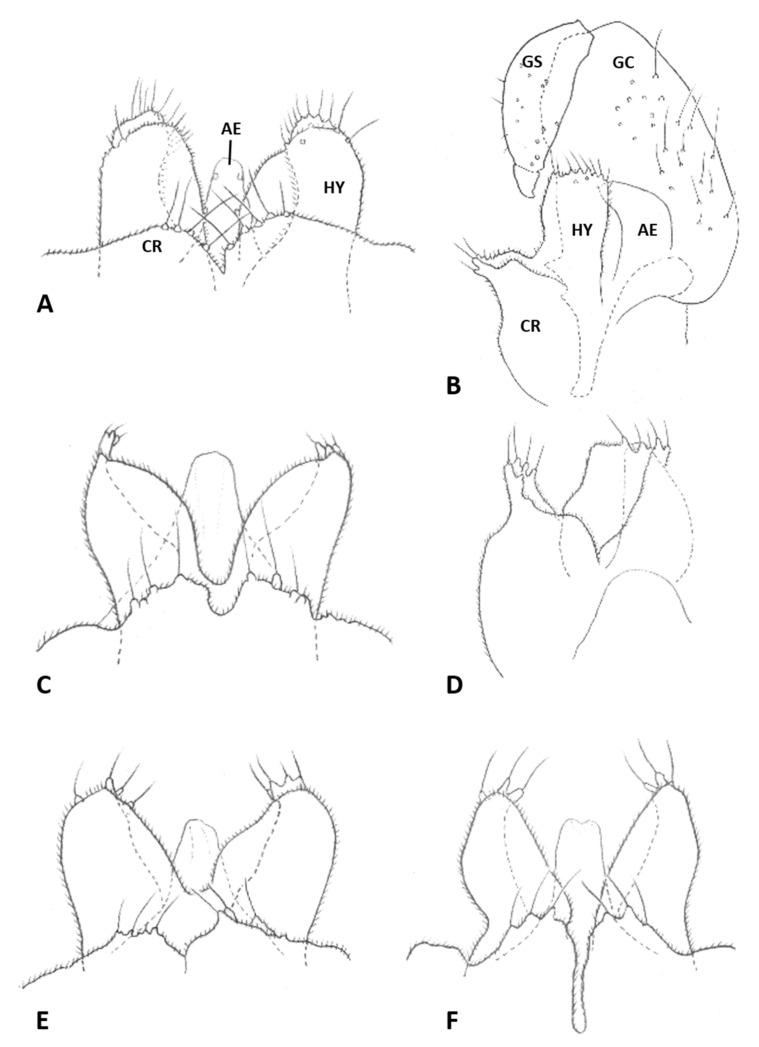
*Asiodiplosis* male terninalia. (**A**). *A. largifica*, dorsal; (**B**). *A. largifica*, lateral (incl. gonopod); (**C**). *A. paradoxa*, dorsal; (**D**). *A. paradoxa*, lateral; (**E**). *A. admirabilis*, dorsal; (**F**). *A. bimoda*, dorsal. AE = aedeagus, CR = cerci, GC = gonocoxite, GS = gonostylus, HY = Hypoproct.

*Male abdomen*: Sclerites usually weakly pigmented, often only in mid-section. Tergites 1–7 with anterior pair of distinct sensory setae, and 1–2 posterior rows of long setae; tergite 8 with anterior pair of sensory setae and few setae posteriorly. Sternites without discernible sensory setae anteriorly, with several long setae on most of surface, not forming clear posterior row. Terminalia (Figure 8A and Figure 9A,B): Gonocoxite robust, wide rectangular at base, gradually narrows distally, completely setulose, with many evenly distributed strong setae. Gonostylus cylindrical, almost same width throughout length, densely setulose, with numerous evenly distributed setae, ending with wide, brush-like tooth apparently composed of large group of fused setae. Gonostyli typically bent anteriorly to lie over gonocoxites. Cerci short and wide, densely setulose, separated only along distal half to form two triangular lobes much shorter than hypoproct, each with 5–6 long, straight apical setae on prominent bases (Figure 9A). Hypoproct completely separated into two long cylindrical lobes, splayed along median margins, arched apically around aedeagus, with each lobe divided by longitudinal groove into wide dorsal section and narrower ventral section, with about 10 strong apical setae. Aedeagus wide at base and tapered towards rounded apex in dorsal view (Figure 9A), cylindrical and dorsally curved in lateral view (Figure 9B), with pair of sensillae on each side.

**Figure 10:**
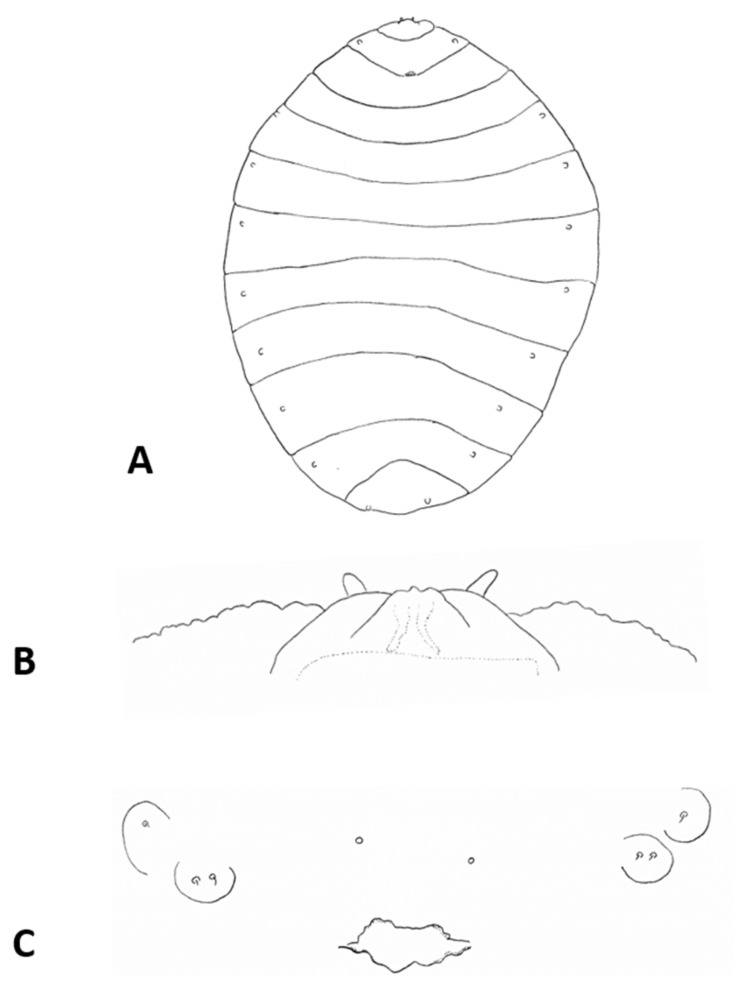
*Asiodiplosis largifica*, larva. (**A**). Habitus, ventral; (**B**). Head; (**C**). Spatula and associated papillae.

**Larva** (third instar) (Figure 10A): Light orange; wide cylindrical, almost spherical. Integument with very delicate rugosity, seen only under SEM. Spiracles dark, situated on elevated projections. Antennae 1.5 times as long as wide; posterolateral apodemes about same length as head capsule (Figure 10B). Spatula rudimentary, comprising small, vaguely defined pigmented patch with only hint of two anterior teeth and no shaft (Figure 10C). Sternal papillae asetose; 3 lateral papillae on each side of spatula, one of which more distant from other two, all with barely visible setae. Other papillae not discernible.

**Figure 11:**
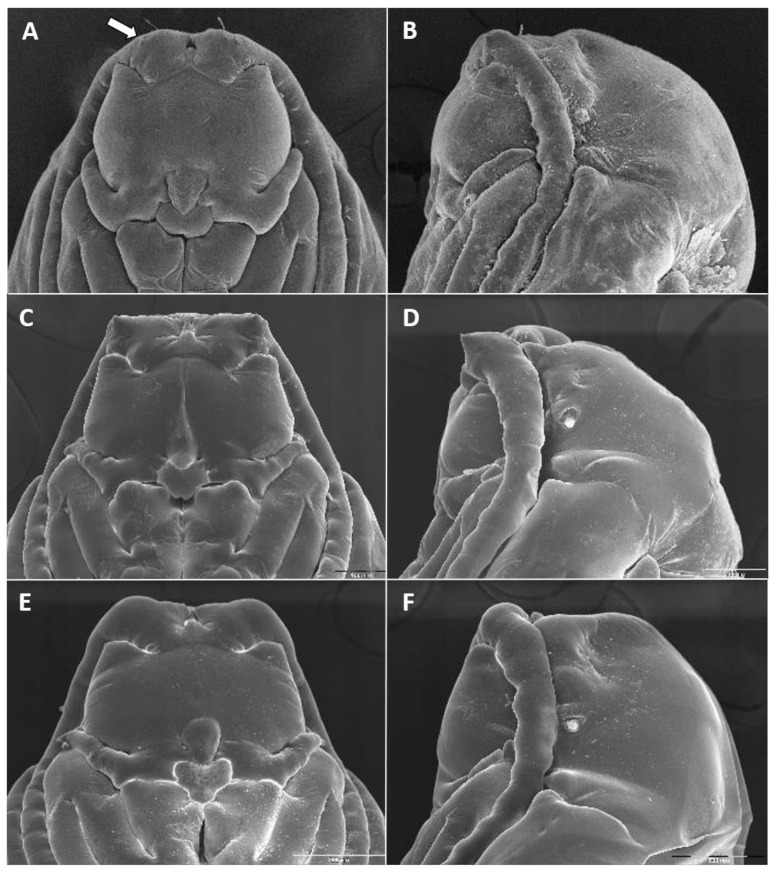
*Asiodiplosis* pupae. (**A**). *A. largifica*, frontal (arrow points to tip on antennal base); (**B**). *A. largifica*, lateral; (**C**). *A. paradoxa*, frontal; (**D**). *A. paradoxa*, lateral; (**E**). *A. admirabilis*, frontal; (**F**). *A. admirabilis*, lateral.

**Pupa** (Figure 11A,B): Light orange. Antennal bases not forming “horns,” with minute tips. Face smooth, without any papillae. Cephalic seta short. Prothoracic spiracle short and blunt, 1–1.5 as long as wide; trachea reaches apex. Abdominal segments evenly covered by tiny spicules.

**Distribution**: This species is common on its main host plant, *C. vermiculatum*, from the southern Golan Heights (around the Sea of Galilee) in the north, to the northern Arava Valley in the south, and the Judaean and Negev deserts in the west. It is particularly common along the Jordan Valley and the Dead Sea. On its second host plant, *C. incanescens*, it was found only in the central Jordan Valley, although the plant is very common in the Negev. The galls of this species may be the ones depicted in Houard [48] from Algeria and Tunisia and attributed to an unidentified gall midge. This would mean that *A. largifica* is widespread at least along the southern and eastern Mediterranean Basin.

**Etymology**: The species name is Latin for bountiful, with reference to the wide distribution and abundance of the galls at almost all times of year.

**Holotype**: **♂**, Israel, Ma’agar Tirza, 32.0667, 35.5061, 23.ii.2021, N. Dorchin, ex bud gall on *Caroxylon vermiculatum*. On permanent microscope slide in Euparal, deposited in SMNHTAU.

**Paratypes**: Ex *Caroxylon vermiculatum:* 1♂, 1♀, Nahal Qumeran, Rt. 90, 31.7375, 35.4597, 19.iii.1995, N. Dorchin; 1♂, 1♀, Nahal Qumeran, 31.7375, 35.4597, 11.v.1995, N. Dorchin; 1♂, 2♀, Nahal Qumeran, 31.7375, 35.4597, 13.ii.1996; 2♂, 1♀, Avenat, 31.6797, 35.4406, 10.vii.1996, N. Dorchin; 1♂, Zomet Mezada, 5 km S, Rt. 90, 31.3131, 35.3833, 27.iv.2014, N. Dorchin; 2♂, Zomet Mezada, 31.3131, 35.3833, 16.ii.2015, N. Dorchin; 8♂, 13♀, HaMeshar, Nahal Terashim, 30.4553, 34.9356, 12.v.2020, N. Dorchin (1♂, 1♀ ZFMK); 4♂, 1♀, Wadi Malha Nature Reserve, 32.0217, 35.4678, 18.viii.2020, N. Dorchin; 4♂, Tomer, Rt. 90, 32.0214, 35.4469, 18.viii.2020, N. Dorchin; 1♂, 2♀, Ma’agar Tirza, 32.0667, 35.5061, 23.ii.2021, N. Dorchin.

Ex *Caroxylon incanescens*: 1♂, 2♀, Zomet Peza’el, 32.0481, 35.4640, 26.ix.1997, N. Dorchin; 11♂, 12♀, Wadi Malha Nature Reserve, 32.0217, 35.4678, 1.vi.2021, Y. Kenigsberg.

**Other material examined**: Ex *Caroxylon vermiculatum* 6 larvae, Enot Zuqim, 31.7156, 35.4514, 15.1.1997, N. Dorchin; 1♂, 2♀, Zomet Mezada, 31.3131, 35.3833, 16.ii.2015, N. Dorchin; 3♂, 8 exuviae, HaMeshar, Nahal Terashim, 30.4553, 34.9356, 12.v.2020, N. Dorchin; 4 larvae, Maagar Tirza, 32.0667, 35.5061, 20.ii.2021, N. Dorchin.

Ex *Caroxylon incanescens*: 1 larva, Wadi Malha Nature Reserve, 32.0217, 35.4678, 1.vi.2021, Y. Kenigsberg; 12 exuviae, Wadi Malha Nature Reserve, 32.0217, 35.4678, 1.vi.2021, Y. Kenigsberg.

**Comments**: To date, 24 species of *Asiodiplosis* were described from *Caroxylon* host plants (originally under *Salsola*) but none of them from *C. vermiculatum* or *C. incanescens*. *Asiodiplosis largifica* belongs to a large group of *Asiodiplosis* species with short setae on segment 9 of the ovipositor, and has the longest ovipositor among the Israeli species (Table 2). The larvae have a rudimentary spatula, contrary to the well-developed spatula or its complete absence in other Israeli species for which larvae are known. The male apical flagellomeres are often fused, and its two-lobed hypoproct is divided longitudinally—two attributes that are seen in many other species in this genus, but these species do not overlap geographically with *A. largifica* and differ in gall morphology and life-history attributes. The two host plants of this species have an Irano-Turanian and Saharo-Arabian distribution, not reaching Central Asia, from which all other *Asiodiplosis* species were described. Taken together with the high level of host specificity seen among Israeli species, we are confident that *A. largifica* is not conspecific with any of the Central-Asian species.

#### 3.2.2. *Asiodiplosis paradoxa* Dorchin, New Species

**Host plants**: *Anabasis setifera* Moq.

**Figure 12:**
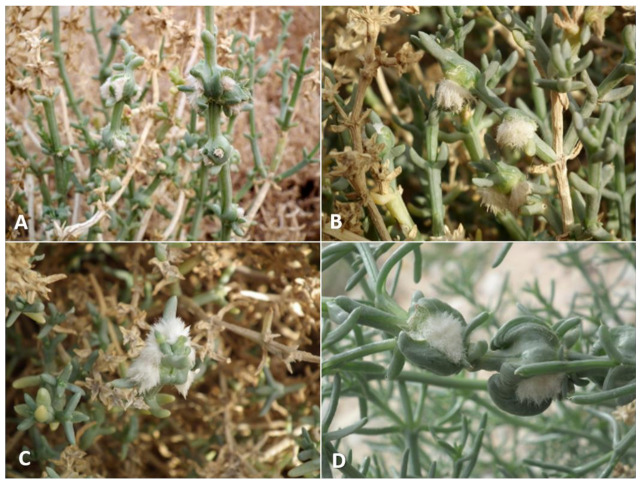
(**A**–**D**) *Asiodiplosis paradoxa* galls on *Anabasis setifera*.

**Gall and biology**: This species induces striking and very common galls in stem joints (Figure 12A–D). The gall constitutes an amorphic enlargement at the base of a joint, accompanied by dense tufts of white hair that seem to burst out of the stem, which makes for a conspicuous sight on the otherwise smooth, glabrous plant. Galls vary considerably in size, ranging from 0.5 to more than 2.5 cm in diameter, and are often grouped together on the same shoot. The size of a gall is not indicative of its age, as small galls may contain a small number of mature larvae or pupae. Each gall contains 1–6 larval chambers embedded in the mass of white hairs immediately above the point at which leaves are attached to the stem. The chambers are rigid capsules composed of several yellowish tapered scales, which appear in the gall when it contains second-instar larvae. First-instar larvae are found in the midst of the green, juicy tissue at the base of the gall without clear chambers. In February and early March, galls contained first-instar larvae, and adults were reared from March to October. Different galls on the same plant may contain larvae of different ages. Clearly this species completes several generations per year, with a peak in spring, and is less common during the summer and fall (July–November), when the plants bear fruits. The galls occasionally contain unidentified moth larvae that feed on the gall tissues as inquilines. Old galls remain on the plants for several months.

**Adult description**: General color: Female pinkish-orange, male grey to pale brown.

*Head*: Antennal flagellomeres 12 in male, 11–12 in female. Male apical flagellomere not fused with preceding, with clearly separated nodes, without apical projection. Circumfilar loops about as long as length of node. Female flagellomeres cylindrical, first 2–3 with slight constriction in mid part, subsequent without clear constriction (Figure 4E), all but two apical flagellomeres with clear necks; apical flagellomere rounded apically, without projection. Frontoclypeal membrane on each side with 5–6 long setae. Palpus 1.5–2 times as long as wide, rounded apically.

*Thorax*: Wing length: 1.28–2.37 mm in female (*n* = 32), 1.45–2.35 mm in male (*n* = 41).

*Female abdomen*: Sclerites usually hardly pigmented. Tergites 1–7 with posterior row of long setae. Ovipositor 5.6–16.9 times as long as tergite 8 (*n* = 27); setae on segment 9 almost as long as segment height (Figure 7A).

*Male abdomen*: Tergites 1–7 with posterior row of setae. Sternites 2–6 with posterior row of setae and few long setae medially; sternites 7–8 with long setae on most of surface. Terminalia (Figure 8B and Figure 9C,D): Gonocoxite hardly narrowed distally. Cerci almost entirely fused, narrowed abruptly at apex to form two small, rounded lobes, each with three strong, straight setae (Figure 9C). Lobes of hypoproct slightly arched around aedeagus, divided longitudinally into wide fusiform dorsal section and narrow, tapered ventral section ending with 3 strong setae on prominent bases (Figure 9C,D). Aedeagus with two longitudinal grooves in dorsal view.

**Larva**: Integument virtually smooth, with very shallow, delicate verrucae. Antennae minute, as long as wide. Spatula absent. Sternal, lateral, and terminal papillae not discernible. Pleural and dorsal papillae asetose.

**Pupa** (Figure 11C,D): Antennal bases form small, tapered horns, pointed ventrally. Head trapezoidal in frontal view.

**Holotype**: ♂, Israel, Nahal Zeruya, Rt. 90, 31.4386, 35.3831, 27.iv.2014, N. Dorchin, ex bud gall on *Anabasis setifera*. On permanent microscope slide in Euparal, deposited in SMNHTAU.

**Paratypes**: 1♂, 1♀, 2 exuviae, En Gedi nature reserve, 31.4549, 35.3942, 19.iii.1995, N. Dorchin; 1♂, 1♀, Nahal Qumeran, Rt. 90, 31.7375, 35.4597, 11.v.1995, N. Dorchin; 2♂, 1♀, Elifaz, 29.7883, 35.0180, 5.iv.1997, N. Dorchin; 1♂, 1♀, Enot Zuqim, 31.7156, 35.4514, 25.iii.2012, N. Dorchin; 3♂, Qalya, Rt. 90, 31.7463, 35.4735, 7.iv.2013, G. Danon; 4♂, 6♀, Enot Zuqim, 31.7156, 35.4514, 17.x.2013, N. Dorchin; 3♂, 2♀, Rt. 206, 3 km S Zomet Rotem, 30.9892, 35.0636, 12.xi.2013, N. Dorchin; 2♂, 1 exuviae, Neot HaKikkar, 30.9519, 35.3635, 27.iv.2014, N. Dorchin; 2♂, 3♀, 8 exuviae, Nahal Zeruya, Rt. 90, 31.4386, 35.3831, 27.iv.2014, N. Dorchin; 6♂, 5♀, 18 exuviae, Mezad Tamar, 31.0275, 35.2417, 27.iv.2014, N. Dorchin (1♂, 1♀ ZFMK); 2♂, 1♀, Arad, 10 km E, Rt. 31, 31.1909, 35.2815, 18.xi.2014, N. Dorchin; 1♂, Nahal Zeruya, Rt. 90, 31.4386, 35.3831, 16.iii.2015, N. Dorchin; 3♂, Nahal Qumeran, Rt. 90, 31.7375, 35.4597, 1.iii.2020, N. Dorchin; 1♂, 1♀, Mezad Tamar, 31.0275, 35.2417, 22.vii.2020, N. Dorchin.

**Other material examined**: 1♀, En Gedi nature reserve, 31.4549, 35.3942, 19.iii.1995, N. Dorchin; 1♀, Elifaz, 29.7883, 35.0180, 5.iv.1997, N. Dorchin; 1♂, 1♀, Enot Zuqim, 31.7156, 35.4514, 25.iii.2012, N. Dorchin; 2♂, 1♀, Enot Zuqim, 31.7156, 35.4514, 17.x.2013, N. Dorchin; 1♂, Nahal Yishay, 31.4841, 35.3969, 17.x.2013, N. Dorchin, G. Danon; 1♂, Rt. 206, 3 km S Zomet Rotem, 30.9892, 35.0636, 12.xi.2013, N. Dorchin; 4 larvae, Nahal Qumeran, Rt. 90, 31.7375, 35.4597, 13.iv.2021, N. Dorchin.

**Distribution**: Very common species throughout the distribution range of its host plant in Israel, from the northern coast of the Dead Sea to the southern Arava Valley in the south and into the Judaean and Negev deserts in the west.

**Etymology**: The species name means strange or unexpected, referring to the striking appearance of the white tufts of hair bursting from the distorted but otherwise smooth, glabrous stems of the host plant.

**Comments**: This is the only Israeli species whose pupae possess distinct antennal horns, and are also distinct in the trapezoidal shape of the head compared to the round pupal heads of other species, in particular, *A. admirabilis* and *A. bimoda* from other *Anabasis* species in Israel. The larvae do not have a spatula, contrary to those of most Israeli species for which larvae are known and in stark contrast to the well-defined spatula in *A. bimoda*. Adults generally resemble those of *A. admirabilis*, but the male antennal flagellomeres do not end with an apical projection, their circumfilar loops are longer, and the ventral, setae-bearing section of the hypoproct is slenderer. The Saharo-Arabian distribution of the host plant, *Anabasis setifera*, its easily recognizable habitus, and the unique appearance of the galls make it highly unlikely that *Asiodiplosis paradoxa* is conspecific with any of the 24 species recorded from Central Asia. Together with *A. largifica*, this is the most-common *Asiodiplosis* species in Israel in terms of gall abundance and adult activity, with adult emergence almost year-round.

#### 3.2.3. *Asiodiplosis admirabilis* Dorchin, New Species

**Host plants**: *Anabasis articulata* (Forssk.) Moq.

**Figure 13:**
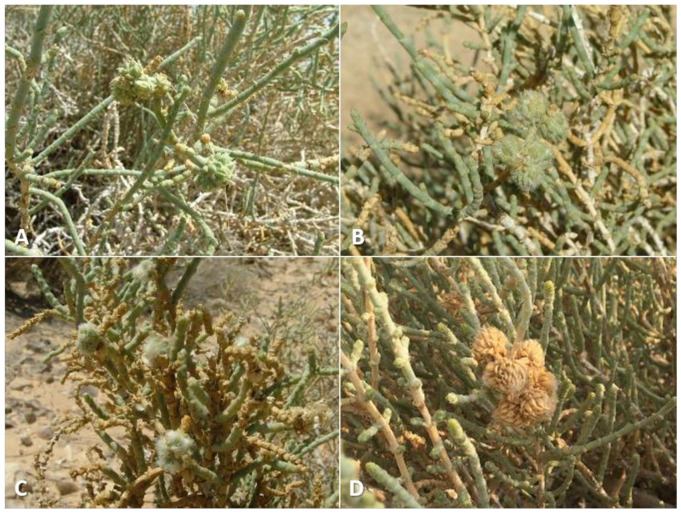
*Asiodiplosis admirabilis* galls on *Anabasis articulata*. (**A**,**B**). Large, spring galls. (**C**). Smaller summer galls. (**D**). Old, dry galls.

**Gall and biology**: This species develops in conspicuous, artichoke-like galls in spring (April) (Figure 13A,B) and in smaller galls in summer and fall (May–September) (Figure 13C). The large spring galls are composed of fleshy scale-like projections accompanied by short, white hairs at the center of the gall and may exceed 2.5 cm in diameter. They are uncommon and difficult to find in most places, but at some sites specific plants harbored tens or even hundreds of galls year after year. Each gall contains 6–10 larval chambers composed of yellowish, tapered scales that are embedded in the white hairs. Old galls remain on the plant for over a year, becoming yellow and eventually grey (Figure 13D). Summer galls are smaller and less conspicuous, usually 5–10 mm in diameter, developing at the bases of joints. They are evident as tufts of white, woolly hairs accompanied by a few green leaf-like scales (Figure 13C) and tend to be found in small groups. Each such gall contains 3–6 larval chambers similar in structure to the larval chambers in spring galls. Adults were notoriously difficult to rear from this host plant compared to the two other *Asiodiplosis* species on *Anabasis* host-plants in Israel. Obviously, this species completes several generations per year in spring and summer. Overwintering probably takes place as first-instar larvae in buds.

**Figure 14:**
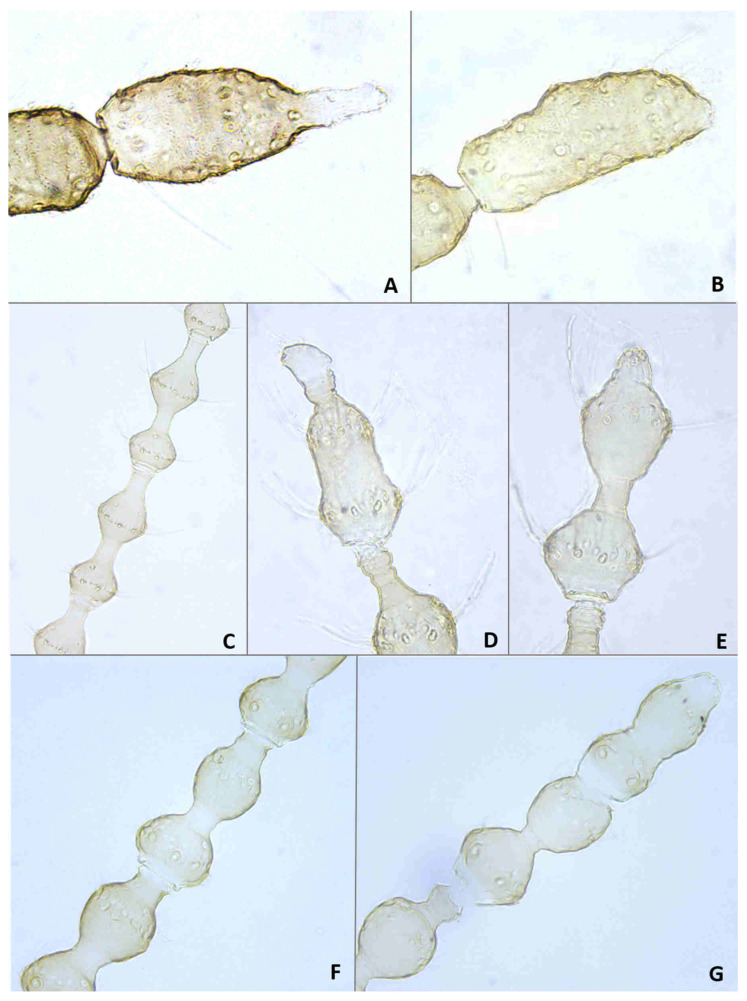
*Asiodiplosis* flagellomeres. (**A**). *A. admirabilis*, male apical flagellomere; (**B**). *A. admirabilis*, female apical flagellomere; (**C**). *A. delicatula*, male flagellomeres 4–6; (**D**,**E**). *A. delicatula*, male apical flagellomere; (**F**). *A. mucronata*, male flagellomeres 4–6; (**G**). *A. mucronata*, male apical flagellomeres.

**Adult description**: *Head:* Antennal flagellomeres 12 in male, 10–11 in female. Necks of male flagellomeres about same length throughout antenna (not successively longer); circumfilar loops about half length of node; apical flagellomere not merged with preceding, sometimes with small elongate projection (Figure 14A). Female flagellomeres, except for apical, all with short necks; length of necks about the same throughout antenna; occasionally, two adjacent flagellomeres partially fused; flagellomeres 2–5 with slight constriction, others without constriction; apical flagellomere composed of 2–3 fused units, sometimes with elongate apical projection (Figure 14B). Frontoclypeal membrane mostly bare, with 1–2 closely adjacent setae on each side. Palpus 1–1.5 as long as wide.

*Thorax*: Wing length: 1.60–2.50 mm in female (*n* = 16), 1.62–2.65 mm in male (*n* = 13).

*Female abdomen*: Sclerites weakly but usually clearly pigmented; pigmentation on tergites often receding on ventral area except around posterior row of setae. Ovipositor 8.58–11.31 times as long as tergite 8 (*n* = 16); setae on segment 9 at most half as long as segment height.

*Male abdomen*: Sclerites usually clearly pigmented; pigmentation on tergites receding on ventral part, often absent along thin line just before posterior line of setae. Terminalia (Figure 9E): Gonocoxite only slightly narrowed distally. Cerci almost completely fused, separated distally to form very short lobes, each with 3–4 long setae apically. Hypoproct almost completely separated into two robust, cylindrical lobes, each divided longitudinally into wide dorsal section and narrower ventral section ending with 3–4 long setae on prominent bases. Aedeagus with two longitudinal grooves in dorsal view.

**Larva**: Not studied.

**Pupa** (Figure 11E,F): Antennal bases not forming horns, without any projections.

**Holotype**: ♂, Israel, Nahal Baraq, Rt. 90, 30.4242, 35.1491, 9.ix.2020, N. Dorchin, ex bud gall on *Anabasis articulata*. On permanent microscope slide in Euparal, deposited in SMNHTAU.

**Paratypes**: 6♀, 1♂, Mamshit, 31.0342, 35.0674, 9.vi.1997, N. Dorchin; 2♂, 2♀, Nahal Lavan, 30.9552, 34.3826, 7.v.1998, N. Dorchin; 1♀, Nahal Shezaf, near Hazeva, 30.7395, 35.2621, 19.iv.2014, N. Dorchin; 4♀, Mezad Tamar, 31.0275, 35.2417, 27.iv.2014, N. Dorchin; 1♂, 1♀, Nahal Ashosh, Rt. 90, 30.5114, 35.1801, 28.iv.2014, N. Dorchin; 3♂, Nahal Baraq, Rt. 90, 30.4242, 35.1491, 9.ix.2020, N. Dorchin; 3♂, 2♀, Nahal Shezaf, near Hazeva, 30.7395, 35.2621, 13.iv.2021, N. Dorchin.

**Other material examined**: 6♀, Mishor Rotem, Rt. 25, 31.0375, 35.1382, 9.vi.1997, N. Dorchin; 1♂, 3 exuviae, Mezad Tamar, 31.0275, 35.2417, 27.iv.2014, N. Dorchin; 3 exuviae, Nahal Baraq, Rt. 90, 30.4242, 35.1491, 9.ix.2020, N. Dorchin; 6 exuviae, Nahal Shezaf, near Hazeva, 30.7395, 35.2621, 13.iv.2021, N. Dorchin. 

**Distribution**: A generally uncommon or sporadic species that is regularly observed in the most arid habitats in Israel, in the Judaean and Negev deserts, and along the Arava valley, particularly in dry riverbeds that experience flash floods once or several times per year. Galls have never been found along the Dead Sea, although the host plant is common in that region.

**Etymology**: The species name is Latin for admirable or remarkable, referring to the occurrence of the galls in the most extreme desert habitats in Israel, sometimes in great numbers.

**Comments**: Adults of this species resemble closely those of *A. paradoxa*, but the apical flagellomeres in both sexes often end with a long projection that is absent in *A. paradoxa*, and the circumfilar loops of the male flagellomeres are shorter. The male hypoproct is somewhat wider in *A. admirabilis* than in *A. paradoxa*, in particular, the ventral, setae-bearing section. Pupae of *A. admirabilis* resemble those of *A. bimoda* but clearly differ from those of *A. paradoxa* in the lack of any projections on the antennal bases compared to the short, pointed antennal horns in *A. paradoxa*. These two species overlap along much of their distribution range but develop on different host plants, and their galls differ profoundly. The scaly and hairy galls of *A. admirabilis* resemble those of *A. bimoda* and those of several *Asiodiplosis* species from *Anabasis* in Central Asia (e.g., *A. anabasidicola* Fedotova, *A. anabasidigemmae* Fedotova, and *A. palpata* Marikovskij [13,46]). Nevertheless, the Saharo-Aarabian distribution of *Anabasis articulata* and differences in life-history attributes between *A. admirabilis* and Central-Asian species make it unlikely that it is conspecific with any of them.

#### 3.2.4. Asiodiplosis bimoda Dorchin, New Species

**Host plants**: *Anabasis syriaca* Iljin.

**Figure 15:**
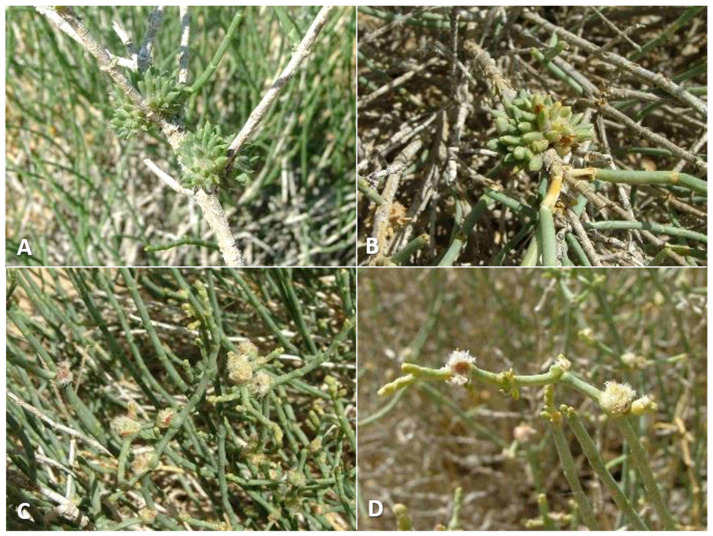
*Asiodiplosis bimoda* galls on *Anabasis syriaca*. (**A**,**B**). Spring galls. (**C**,**D**). Summer galls.

**Gall and biology**: This species induces two types of galls at different times of the year. Spring galls (March–April) are large and very conspicuous cone-like structures, up to 2 cm in diameter and composed of fleshy leaf-like projections accompanied by long, white hairs (Figure 15A,B). In late March, such galls contain third-instar larvae, and adults emerge from them in April. In summer and fall, a much smaller type of gall develops on the same plants in the form of a small tuft of short, white hairs in slightly swollen stem joints, causing the joint to bend and distort (Figure 15C,D). Adults emerge from these galls from June to October. Both types of galls contain several larval chambers—5–10 in spring galls and 1–6 in summer galls—that are embedded in the wooly hairs, composed of yellowish tapered scales at the base of the gall. Both types of galls can be found on the plants during summer, but the larger spring galls are already empty at that time. They remain on the plant for over a year, turning yellow and eventually grey as they dry up. Obviously, this species completes several generations per year in spring and summer. Overwintering probably takes place as first-instar larvae in dormant buds.

**Adult description**: *Head*: Antennal flagellomeres 12 in male, 11 in female. Necks of male flagellomeres about same length throughout antenna; circumfilar loops about half length of node; apical flagellomere not fused with preceding, occasionally with small apical projection. Female flagellomeres with short necks, all but apical three with median constriction; apical flagellomere without distal projection. Frontoclypeal membrane with 5–7 setae on each side. Palpus usually 1.0–1.5 times as long as wide, infrequently longer, up to 4.0 times as long as wide.

*Thorax*: Wing length: 1.75–2.68 mm in female (*n* = 41), 2.12–2.86 mm in male (*n* = 44).

*Female abdomen*: Ovipositor 9.00–15.13 as long as tergite 8 (*n* = 36); setae on segment 9 0.5–0.7 times as long as segment height.

*Male abdomen*: Sclerites usually clearly pigmented. Terminalia (Figure 9F): Cerci separated to base, each with 3 long apical setae. Hypoproct divided longitudinally into wide-dorsal and thinner-ventral sections, thinner section with 3 long apical setae on prominent bases. Aedeagus with small apical cleft.

**Figure 16:**
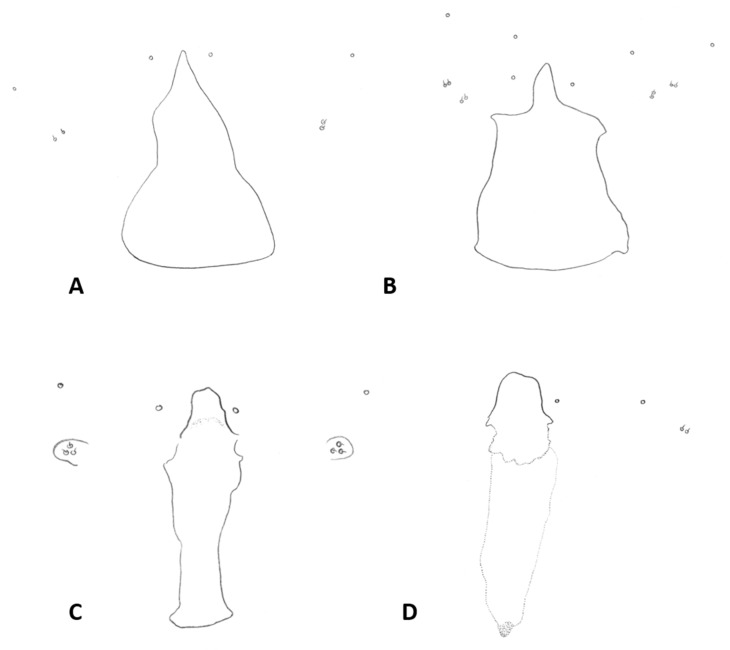
*Asiodiplosis* larval spatulae and associated papillae. (**A**). *A. bimoda*; (**B**). *A. delicatula*; (**C**). *A. stellata*; (**D**). *A. pillosaeconspicua* (papillae shown only on right side).

**Larva** (third instar): Varies greatly in size; some mature larvae collected at the same locality and date were tenth the size of others. Spatula (Figure 16A) well-developed, with very wide shaft, narrowed anteriorly into single triangular tooth. Sternal papillae asetose; 3 three lateral papillae on each side, arranged in group of two setose and one distant asetose papilla. Other papillae not discernible.

**Figure 17:**
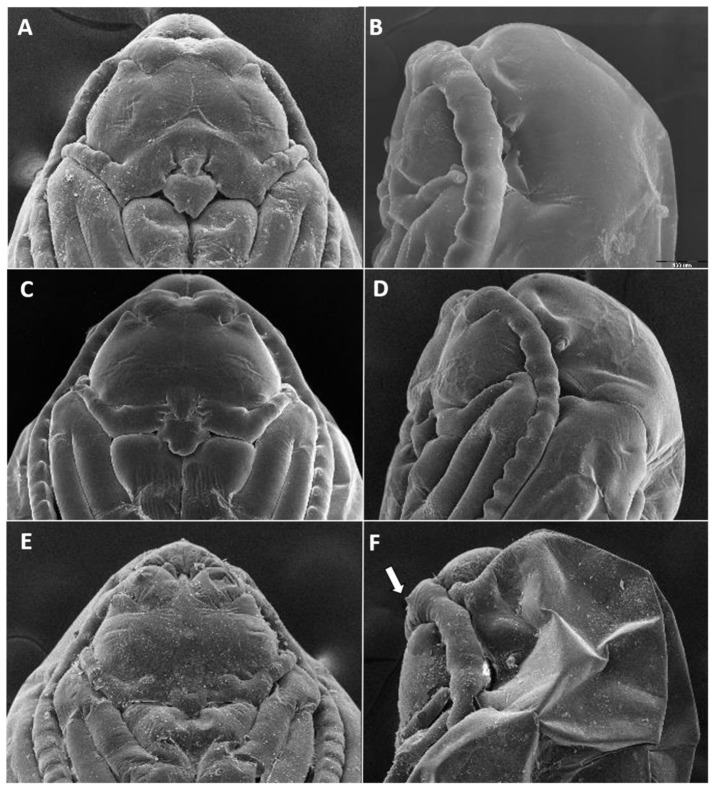
*Asiodiplosis* pupae. (**A**). *A. bimoda*, frontal; (**B**). *A. bimoda*, lateral; (**C**). *A. delicatula*, frontal; (**D**). *A. delicatula*, lateral; (**E**). *A. pillosaeconspicua*, frontal; (**F**). *A. pillosaeconspicua*, lateral (arrow points to tip on antennal base).

**Pupa** (Figure 17A,B): Antennal bases smooth, without any projections.

**Holotype**: ♂, Nahal Zin, Rt. 40, Zomet haRuhot, 5 km N, 30.7092, 34.7839, 16.vi.2014, N. Dorchin & I. Hayon, ex bud gall on *Anabasis syriaca*. On permanent microscope slide in Euparal, deposited in SMNHTAU.

**Paratypes**: 3♂, 3♀, Nahal Ye’elim, Rt. 31, 31.2388, 35.2357, 19.iv.1995, N. Dorchin; 2♂, 2♀, Ezuz, 30.8021, 34.4661, 17.iv.1998, A. Freidberg; 2♂, 2♀, Nevatim, 31.2156, 34.8799, 14.x.1998, N. Dorchin; 1♂, 1♀, Nahal Zin, Rt. 40, 30.7092, 34.7839, 27.vi.2012, N. Dorchin; 7♂, 6♀, Nahal Boqer, Rt. 40, 30.9096, 34.7779, 28.iii.2013, G. Danon; 8♂, 8♀, Nahal Ye’elim, Rt. 31, 31.2388, 35.2357, 17.iv.2013, N. Dorchin; 1♂, 1♀, Nahal Ye’elim, Rt. 31, 31.2388, 35.2357, 9.iv.2014, N. Dorchin; 5♂, 5♀, El Atrash, Rt. 31, nr. Hura, 31.2693, 34.9519, 11.vi.2014, Y. Sapir; 6♂, 2♀, Zomet haNegev, 31.0666, 34.8381, 21.iv.2014, G. Danon; 5♂, 6♀, Nahal Zin, Rt. 40, Zomet haRuhot, 5 km N, 30.7092, 34.7839, 16.vi.2014, N. Dorchin & I. Hayon; 1♂, 1♀, Nahal Zin, Rt. 40, 30.7092, 34.7839, 22.vii. 2020, N. Dorchin; 8 larvae, Nahal Ye’elim, Rt. 40, 31.2388, 35.2357, 25.iii.2021, N. Dorchin.

**Other material examined**: 2♂, 3♀, Nahal Boqer, Rt. 40, 30.9096, 34.7779, 28.iii.2013, G. Danon; 1♀, Nahal Ye’elim, Rt. 31, 31.2388, 35.2357, 17.iv.2013, N. Dorchin; 16 exuviae, Zomet HaNegev, 31.0666, 34.8381, 21.iv.2014, G. Danon.

**Distribution**: This species is found in high elevations in the Negev desert, wherever its host plant, *Anabasis syriaca*, occurs.

**Etymology**: The species name refers to the two types of galls it induced at different times of the year.

**Figure 18:**
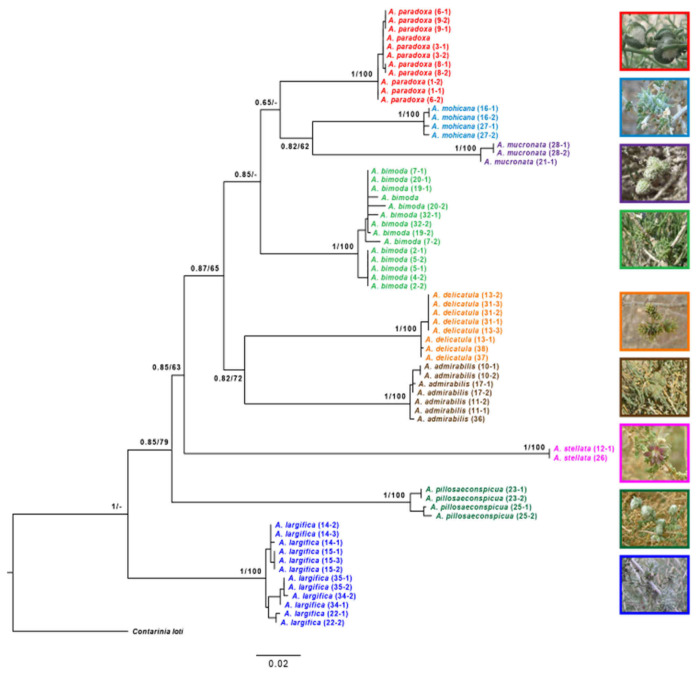
A maximum likelihood (IQtree) phylogenetic tree based on concatenated mitochondrial COI and 16S sequences of 64 ingroup taxa, with *Contarinia loti* as an outgroup (details and GenBank accession numbers given in Table 1). Support values for main clades (>0.65 posterior probabilities and >60% ML bootstrap values) are shown next to nodes. Species colors correspond to the respective gall images on the right.

**Comments**: Adults, pupae, and galls of this species resemble those of *A. admirabilis* from *Anabasis articulata*, but the two species do not overlap in Israel (Figure 1) and are clearly distinct from each other genetically (Figure 18). See comments under *A. admirabilis* and *A. paradoxa* for more differences between them and other species from *Anabasis* from Central Asia.

#### 3.2.5. *Asiodiplosis delicatula* Dorchin, New Species

**Host plants**: *Haloxylon persicum* Bunge.

**Figure 19:**
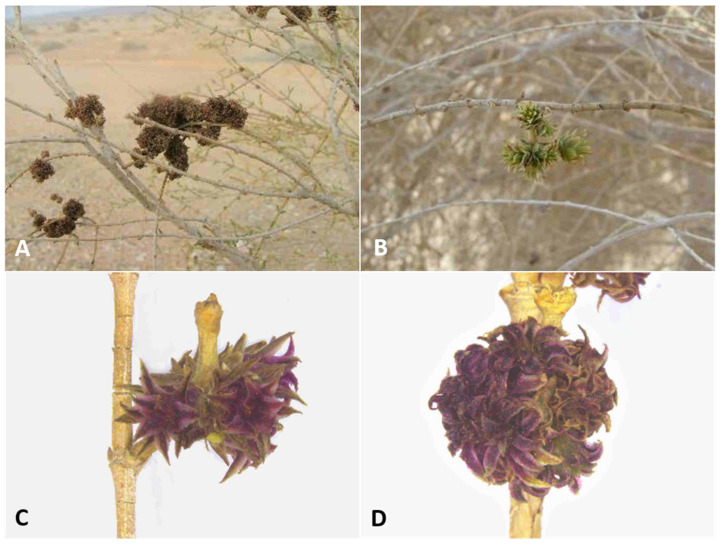
*Asiodiplosis delicatula* galls on *Haloxylon persicum*. (**A**) clusters of galls on old branches. (**B**–**C**) closeup of small gall clusters. (**D**) closeup of large cluster.

**Gall and biology**: This species develops in rosette-like bud galls, about 3 mm in diameter (Figure 19B,C). Very often several galls are grouped in 5–10 units to form spherical aggregations, 6–8 mm in diameter (Figure 19A,D). The gall is composed of elongate, tapered leaf-like scales that are bent outwards except for 2–3 scales at the center of the gall, which are closely appressed to form a rigid cone with a cup-like bottom.

The leaf-like scales are green to brownish or magenta, and the galls stand out on the background of the plant’s leafless stems despite their small size. A single plant can harbor hundreds of galls mostly on young green shoots but also on older, woody shoots. Empty galls dry up, turn yellow, and stay on the plant for several weeks. The large, almost-spherical larvae fill up the larval chamber, as do their hymenopteran parasitoids. This species has only one generation per year, from January to February, when galls are very common at almost all sites where the host plant occurs in Israel. Galls found on plants in later months were empty.

**Adult description**: *Head*: Antennal flagellomeres 12 in male, 11 in female. Male flagellomeres with proximal node larger than distal node, rhomboidal rather than spherical (Figure 14C); distal necks successively longer; circumfilar loops as long as node (Figure 4B); apical flagellomere often with short-to-medium-sized projection (Figure 14D,E). Female flagellomeres successively shorter, except apical flagellomere longer than preceding; proximal 6–7 flagellomeres with slight constriction and short necks; distal flagellomeres without clear constriction and necks. Frontoclypeal membrane with 3–8 long setae on each side. Palpus usually spherical, as long as wide, occasionally 1.5–2.0 times as long as wide.

*Thorax*: Wing length: 2.54–2.94 mm in female (*n* = 23), 2.76–3.04 mm in male (*n* = 20).

*Female abdomen*: Sclerites usually clearly pigmented, pigmentation receding on antero-ventral part. Ovipositor 6.46–16.21 times as long as tergite 8 (*n* = 21); segment 9 with dense cover of very long hair-like setae, denser and longer along proximal half; setae much longer than ovipositor width (Figure 7C).

**Figure 20:**
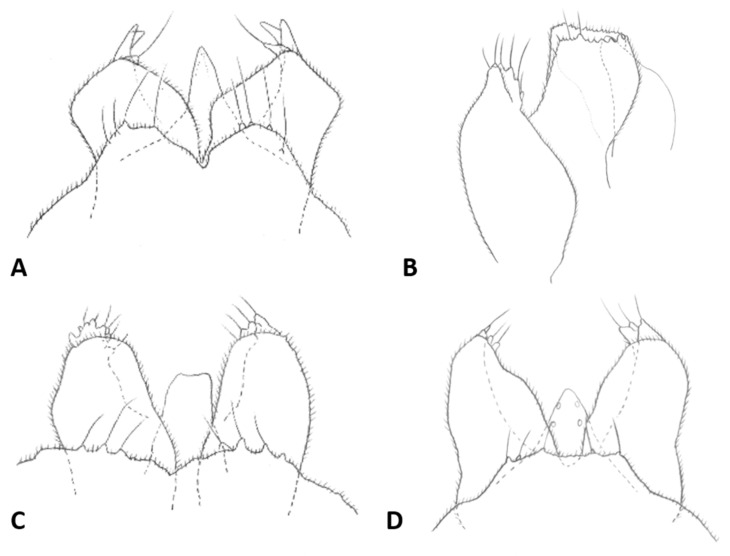
*Asiodiplosis* male terminalia. (**A**). *A. delicatula*, dorsal; (**B**). *A. delicatula*, lateral; (**C**). *A. stellata*, dorsal; (**D**). *A. mucronata*, dorsal.

*Male abdomen*: Sclerites as in females. Terminalia (Figure 20A,B): Cerci separated apically to form two short, rounded lobes, each with 4 strong setae apically. Hypoproct separated almost to base into two lobes, each divided longitudinally into massive, rectangular dorsal lobe and thinner ventral lobe with three tapered apical projections bearing long setae.

**Larva** (third instar): Integument virtually smooth. Spatula (Figure 16B) conspicuous and strongly pigmented, with short and wide rectangular shaft and single, tapered tooth. Sternal papillae asetose, lateral papillae minute, setiform, usually arranged in two pairs of two, but occasionally one papilla in more-distal group lost or three papillae grouped together in proximal group. Terminal papillae, 3–4 on each side, asetose, barely visible.

**Pupa**: Antennal bases smooth, without any projections.

**Holotype**: ♂, Israel, Lotan, 29.9811, 35.0857, 10.ii.2021, N. Dorchin, ex bud gall on *Haloxylon persicum*. On permanent microscope slide in Euparal, deposited in SMNHTAU.

**Paratypes**: 2♂, 3♀, 2 larvae, Yotvata, 29.8841, 35.0771, 9.i.1996, N. Dorchin; 4♂, 3♀, Yotvata salt marsh, 29.8359, 35.0459, 12.ii.2013, N. Dorchin; 7♂, 8♀, En Yahav, 30.6450, 35.2085, 12.ii.2013, N. Dorchin (1♂, 1♀ ZFMK); 2♂, 3♀, 5 larvae, Lotan, 29.9811, 35.0857,10.ii.2021, N. Dorchin; 3♂, 3♀, Holot Kasuy, 29.9839, 34.9785, 11.ii.2021, N. Dorchin, M. Spodek.

**Other material examined**: 1♂, 2 exuviae, Yotvata, 29.8841, 35.0771, 9.i.1996, N. Dorchin; 3♀, 3 exuviae, En Yahav, 30.6450, 35.2085, 12.ii.2013, N. Dorchin.

**Distribution**: Common along the Arava Valley.

**Etymology**: The species is named after its small, soft-leaved galls, which are more delicate than galls of other *Asiodiplosis* species in Israel.

**Comments**: This species is the only representative in Israel of a group within *Asiodiplosis* in which segment 9 of the ovipositor is covered by very long hair-like setae (e.g., *A. botryoidea* (Fedotova), *A. densipila* (Marikovskij), *A. festinans* (Marikovskij), and *A. rhaphidophytonis* (Fedotova) [6,8,49,50]. It is also readily distinguishable from other Israeli species in the rectangular lobes of the male hypoproct and the conspicuous projection of the apical antennal flagellomere in both sexes. The “hairy” ovipositor and rectangular male hypoproct are often, but not always, correlated, and are common among the 18 *Asiodiplosis* species from *Haloxylon*, all from Central Asia. Only two of these species—*A. floripara* (Mamaev) and *A. saxauli* (Kaplin)—were recorded from *Haloxylon persicum*, with the remaining from *H. ammodendron* (C.A.Mey.) Bunge ex Fenzl (=*H. aphyllum*). Mamaev [16] did not include any Figures in the description of *A. floripara* from Uzbekistan, but the description of the galls and the male hypoproct do not fit those of *A. delicatula*. *Asiodiplosis saxauli*, described by Kaplin [50] from Turkmenistan, has male flagellomeres that are almost devoid of necks and ovipositor with sparse, short setae. These attributes, together with the description of the galls, leave no doubt that *A. saxauli* is distinct from *A. delicatula*. *Asiodiplosis vernalis* Marikovskij, described from *H. ammodendron* in Kazakhstan and Turkmenistan [6], is rather similar morphologically to *A. delicatula*, but its galls (depicted by Mamaev [51]) are scaly aggregations rather than soft-leaved rosettes as those of *A. delicatula*.

#### 3.2.6. *Asiodiplosis stellata* Dorchin, New Species

**Host plants**: *Caroxylon tetrandrum* (Forssk.) Akhani and Roalson.

**Figure 21:**
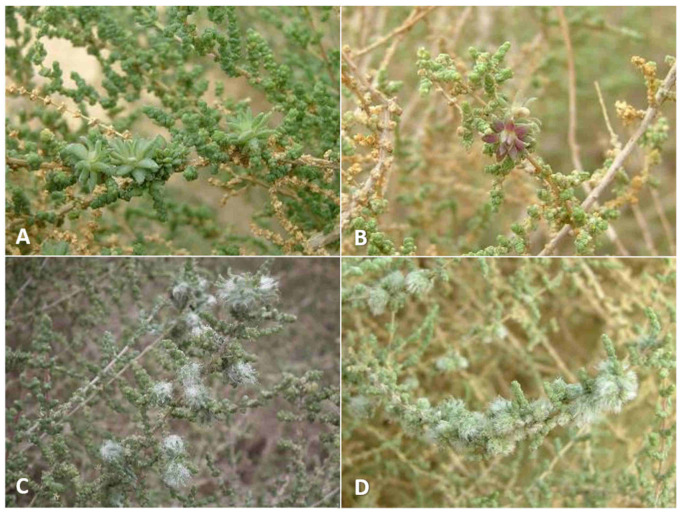
*Asiodiplosis* galls on *Caroxylon tetrandrum*. (**A**,**B**). *A. stellata*; (**C**,**D**). *A. pillosaeconspicua*.

**Gall and biology**: This species induces elegant galls reminiscent of waterlily flowers, 5–15 mm in diameter. The size of a gall does not reflect its age, as small galls contained mature larvae or pupae. The gall is composed of several fleshy, triangular leaves that are much larger and that differ considerably in shape from the normal, scaly leaves of the host plant (Figure 21A,B), and is accompanied by very few hairs. Despite this, the galls are rather cryptic due to their infrequent occurrence and the fact that most of them are green. Some galls that are pinkish to dark magenta are easier to locate. Old galls turn yellow and remain on the plants for several months. Each gall contains a single very large larva that fills the round and rigid central chamber completely. Viable galls were found only from mid-January to March, suggesting that this species has a single generation per year.

**Adult description**: A strikingly large and dark gall midge. General color of both sexes tan-brown (including wings), female abdomen dark red.

**Figure 22:**
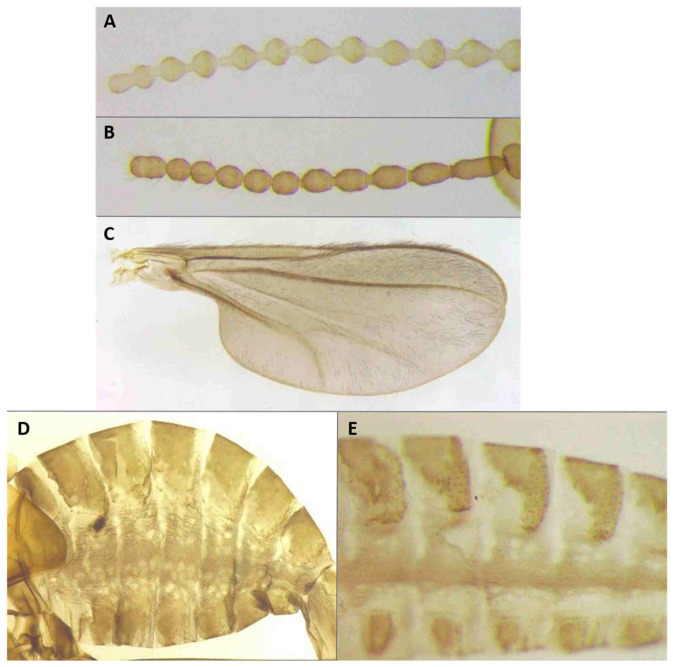
*Asiodiplosis stellata*. (**A**). Male antenna; (**B**). Female antenna; (**C**). Wing; (**D**). Female abdomen; (**E**). Male abdomen.

*Head*: Antennal flagellomeres12 in male, 11–12 in female. necks of male flagellomeres successively longer (Figure 22A); apical flagellomere without neck between nodes; circumfilar loops as long as node. Female flagellomeres (Figure 22B) successively shorter, often without constriction; proximal flagellomeres cylindrical, distal flagellomeres almost spherical. If 11, apical flagellomere longer than preceding, composed of two merged units; necks same length to flagellomere 6 or 7, shorter thereafter. Frontoclypeal membrane on each side with 15–20 setae in male, 5–12 setae in female. Palpus 1–2 times as long as wide.

*Thorax*: Wing (Figure 22C): tan-brown; length: 1.58–2.10 mm in female (*n* = 16), 2.62–3.56 mm in male (*n* = 6).

*Female abdomen* (Figure 22D): tan-brown; sclerites usually strongly pigmented, tergite pigmentation receding on antero-ventral part; posterior row of setae extending into 2–3 rows on ventral part of tergites 1–7. Sternites with posterior row of setae and numerous setae elsewhere, posterior sternite more setose than anterior ones. Pleura densely covered by minute dark spicules except for bare spherical areas, creating white mottled pattern. Ovipositor 9.3–13.0 times as long as tergite 8; setae on segment 9 about 0.3 times as long as ovipositor height.

*Male abdomen* (Figure 22E): Sclerites usually strongly pigmented. Tergite pigmentation receding on antero-ventral part, tergites with 3–4 lines of setae occupying posterior quarter to half of tergite length. White mottled pattern on pleura weaker than in female. Terminalia (Figure 20C): Cerci shrot, almost completely fused, separated by very shallow notch apically, each with 4 long setae. Hypoproct separated into two cylindrical lobes, not arched towards aedeagus, weakly grooved into wider-dorsal and slightly narrower-ventral sections, each lobe with several strong setae pointed apically. Aedeagus very wide, truncate apically.

**Larva** (third instar): Bright orange. Head short and wide, posterolateral apodemes as long as head capsule, antennae tapered, about twice as long as wide. Spatula (Figure 16C) well-developed, with single blunt tooth and long shaft, widest just proximal to tooth. Sternal papillae asetose; lateral papillae in one group of 3 on each side, with tiny setae; occasionally, only two lateral papillae in group, or two grouped and one set further away; arrangement may vary between two sides of same individual.

**Figure 23:**
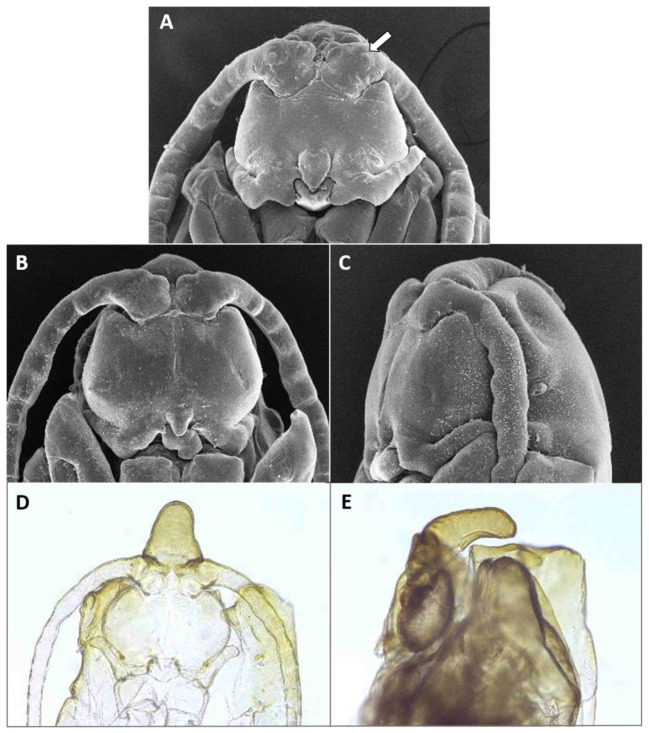
*Asiodiplosis* pupae. (**A**). *A. stellata*, frontal (arrow points to tip on antennal base); (**B**). *A. mucronata*, frontal; (**C**). *A. mucronata*, lateral; (**D**). *A. mohicana* exuviae, frontal; (**E**). *A. mohicana* exuviae, lateral.

**Pupa** (Figure 23A): Antennal bases with minute tapered projection. 

**Holotype**: ♂, Israel, Nahal Zeruya, 31.4386, 35.3831, Rt. 90, 3.iii.2015, G. Danon, ex bud gall on *Caroxylon tetrandrum*. On permanent microscope slide in Euparal. Deposited in SMNHTAU.

**Paratypes**: 5♀, 5 larvae, En Gedi, Rt. 90, 31.4549, 35.3942, 25.1.1996, N. Dorchin; 2♀, 2♂, En Gedi, Rt. 90, 31.4549, 35.3942, 13.ii.1996, N. Dorchin; 1♀, 3 larvae, En Gedi, Rt. 90, 31.4549, 35.3942, 16.i.1997, N. Dorchin; 1♂, Nahal Zeruya, Rt. 90, 31.4386, 35.3831,3.iii.2015, G. Danon; 1♀, HaMeshar, 30.4553, 34.9356, 20.ii.2020, N. Dorchin; 1 larva, Nahal Zeruya, Rt. 90, 31.4386, 35.3831, 16.iv.2020, N. Dorchin, O. Segal; 1 larva, Nahal Zeruya, Rt. 90, 31.4386, 35.3831, 2.iii.2021, Y. Kenigsberg, O. Fischer; 5♀, 2♂, Nahal Zeruya, Rt. 90, 31.4386, 35.3831, 14.ii.2021, O. Fischer, Y. Kenigsberg, R. Raz.

**Other material examined**: 2♀, En Gedi, Rt. 90, 31.4549, 35.3942, 25.i.1996, N. Dorchin; 3 exuviae, Nahal Zeruya, Rt. 90, 31.4386, 35.3831, 14.iii.2021, O. Fischer, Y. Kenigsberg, R. Raz.

**Distribution**: Rare species that was regularly found in small numbers at only one locality in the Dead Sea area and on one occasion in the Negev Desert.

**Etymology**: This species is named after its galls, which are reminiscent of small stars.

**Comments**: This species stands out among other Israeli *Asiodiplosis* species for its large size and conspicuously dark color. It is also unique for the barrel-shaped female flagellomeres that lack median constrictions in some individuals and the tergite setation, consisting of 3–4 lines of posterior setae as opposed to 1–2 lines in other species. It develops on the same host plant with *A. pillosaeconspicua,* but its less-common galls (Figure 21A,B) are easily distinguishable from the hairy galls of *A. pillosaeconspicua* (Figure 21C,D). In the laboratory, the heavy adults were not inclined to fly and were found stumbling around on the bottom of the rearing cage.

#### 3.2.7. *Asiodiplosis pillosaeconspicua* Dorchin, New Species

**Host plants**: *Caroxylon tetrandrum*

**Gall and biology**: This species develops in compact, hairy galls, 1.0–1.5 cm long and 1.0 cm wide, composed of flat, elongate, green leaves that differ markedly from the normal scaly leaves of the plant, and a mass of woolly white hairs between and around the leaves (Figure 21C,D). Galls are very common and are usually found in groups along the same shoots. Each gall contains 1–5 larvae in chambers composed of yellowish, triangular scales at the base of the gall. Adults were reared from mid-February to late June, but some galls dissected in March and April contained first-instar larvae. Therefore, this species appears to complete several generations in spring and early summer, whereas, in other times of the year, it is probably present as inactive first-instar larvae in the buds. Old galls remain on the plant for at least several weeks. These galls are much more common than those of *A. stellata* on the same host plant, and differ from them clearly in structure and pilosity (compare Figure 21A and Figure 21C).

**Adult description**: *Head*: Antennal flagellomeres 12 in male, 11–12 in female. Male flagellomeres with successively longer necks, proximal node sphereical, distal node oval. First two flagellomeres fused, first 2–4 often without necks between two nodes; apical flagellomere oval, without division into two nodes; circumfilar loops about half node length (Figure 4C). Female flagellomeres 1–8 or 1–10 with short necks; when 11, apical flagellomere about twice as long as preceding, without apical projection. Palpus 1–1.5 times as long as wide. Frontoclypeal membrane with 2–4 setae on each side in female, 3–6 in male.

*Thorax*: Wing length: 1.26–2.15 mm in female (*n* = 11), 1.70–2.45 mm in male (*n* = 24).

*Female abdomen*: Sclerites usually clearly pigmented. Tergites 1–7 with one posterior row of setae, reduced to 2–4 setae on lower part of distal tergites. pleura with mottled pattern created by bare spherical areas on background of minute dark spicules. Ovipositor 6.2–13.6 times as long as tergite 8 (*n* = 9); setae on segment 9 about half length of ovipositor height.

*Male abdomen*: Sclerites usually clearly pigmented. Tergite setation and pleura as in female. Terminalia (Figure 8D): Virtually similar to those of *A. largifica*. Cerci almost completely fused, separated by shallow notch apically, each with 3 long apical setae. Hypoproct separated to base into two cylindrical lobes, weakly grooved into wider dorsal section and slightly narrower ventral section; each lobe with 6–7 long setae apically. Aedeagus wide, rounded to truncate apically.

**Larva** (third instar): Spatula (Figure 16D) with clearly pigmented, nearly rectangular proximal lobe and weakly pigmented shaft. Lateral papillae in group of 2–3 on each side, with tiny setae.

**Pupa** (Figure 17E,F): Antennal bases with minute tapered projection.

**Holotype**: ♂, Israel, HaMeshar, Rt. 40, 30.4553, 34.9356, 27.iii.2020, N. Dorchin, ex bud gall on *Caroxylon tetrandrum*. On permanent microscope slide in Euparal, deposited in SMNHTAU.

**Paratypes**: 1♀, Qalya, 31.7463, 35.4735, 15.i.1997, N. Dorchin; 1♂, En Avedat, 30.8337, 34.7722, 18.ii.2015, N. Dorchin; 1♀, 4♂, HaMeshar, 30.4553, 34.9356, 20.ii.2020, N. Dorchin; 3♀, 9♂, HaMeshar, Rt. 40, 30.4553, 34.9356, 27.iii.2020, N. Dorchin.

**Other material examined**: 2 larvae, Qalya, 31.7463, 35.4735, 15.i.1997, N. Dorchin; 2 exuviae, Nahal Zeruya, Rt. 90, 31.4386, 35.3831, 27.iv.2014, N. Dorchin; 1♂, En Avedat, 30.8337, 34.7722, 18.ii.2015, N. Dorchin; 6♀, 8♂, HaMeshar, 30.4553, 34.9356, 20.ii.2020, N. Dorchin; 1 larva, Nahal Qumeran, 31.7375, 35.4597, 2.iii.2021, N. Dorchin.

**Distribution**: Common along the Dead Sea and the Negev and Judaean deserts.

**Etymology**: This species is named after its hairy, gregarious galls, which are common and easily detected.

**Comments**: This species is rather similar morphologically to *A. stellata*, which develops on the same host plant, but is notably smaller and not as dark, and the galls of the two species are readily distinguishable. The two species share the white mottled pattern on the abdominal pleura, although it is much more pronounced in *A. stellata*, as well as the structure of male terminalia and the pupal morphology. Differences among them include the shorter circumfilar loops on the male flagellomeres in *A. pillosaeconspicua*, the almost-spherical rather than cylindrical distal female flagellomeres in *A. stellata*, the more-setose tergites in *A. stellata*, and the quadrate rather than tapered anterior lobe of the spatula in *A. pillosaeconspicua*.

#### 3.2.8. *Asiodiplosis mucronata* Dorchin, New Species

**Host plants**: *Cornulaca monacantha* Delile.

**Figure 24:**
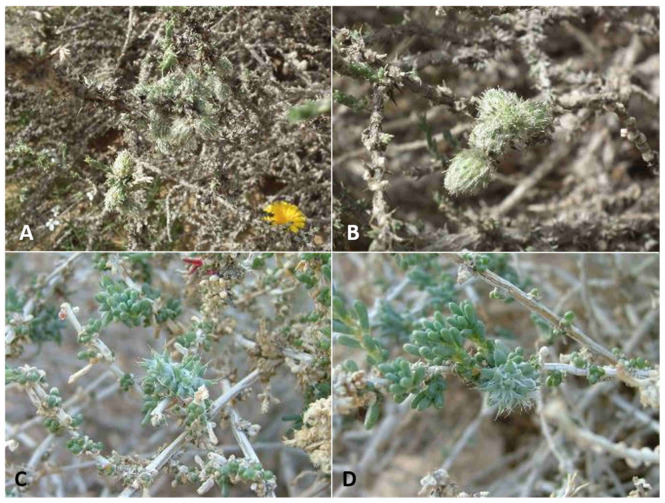
*Asiodiplosis* galls. (**A**,**B**). *A. mucronata* on *Cornulaca monacantha*; (**C**,**D**). *A. mohicana* on *Agathophora allopecuroides*.

**Gall and biology**: This species develops in scaly, spiny artichoke-shaped galls (Figure 24A,B) that are locally very common. Fully-grown galls are 5–7 mm in diameter, often in groups of 2–4 galls together. They are composed of scores of small, tapered scale-like leaves, the internal ones thinner and softer, the external ones wider, more rigid, and concave, with a formidable apical thorn. The scales are mixed with long, white hairs. Each gall contains a single central larval chamber composed of closely appressed scales. Although the galls are found by the thousands, it is extremely difficult to rear the gall midges from them because the eclosion period is very short and galls that are collected too early do not yield adults. There is only one generation per year, with adult emergence in the first half of May. Galls that were sampled in March were fully developed but contained tiny first-instars; galls sampled in April did not yield adults, and galls collected after mid-May were already empty.

**Adultdescription**: *Head*: Antennal flagellomeres 12 in male, 11–12 in female. Male flagellomeres with very short necks between nodes and short and wide distal necks, slightly longer successively along antenna; two apical flagellomeres fused; apical flagellomere without separation to two nodes, sometimes with small apical projection; circumfilar loops about half length of node. Female flagellomeres about same length or slightly successively shorter along antenna. except longer first and last flagellomeres; when 11, last flagellomere composed of two merged units, sometimes with small apical projection; all flagellomeres with median constriction and wide necks, neck about same length throughout antenna. Palpus 1.5–3.0 times as long as wide, often widest distally. Frontoclypeal membrane with single long seta on each side.

*Thorax*: Wing length: 2.57–3.02 mm in female (*n* = 10), 2.51–3.02 mm in male (*n* = 10).

*Female abdomen*: Posterior row of setae on tergites expanded into 2–3 rows along ventral half of tergite. Ovipositor as long as tergite 8 (*n* = 7). Setae on segment 9 0.5–1 times as long as segment height.

*Male abdomen*: Terminalia (Figure 20D): Gonostylus with slight shallow depression along proximal margin close to base. Cerci completely fused, sometimes with slight, shallow depression apically and 2–3 long setae on each side. Hypoproct divided into two lobes almost to base, parallel-sided laterally, narrowed abruptly to rounded notch medially, slightly arched distally; each lobe divided longitudinally into narrow ventral section ending with 3–4 long setae and somewhat wider dorsal section. Aedeagus wide and truncate apically.

**Larva** (third instar): Spatula absent. Discernible papillae include asetose sternals and pleurals, and two slightly protruding, asetose terminal papillae.

**Pupa** (Figure 23B,C): Antennal bases smooth, without any projections. 

**Holotype**: ♂, Israel, Sede Halamish, 30.9219, 34.4053, 3.v.2020, A. Dorchin, ex bud gall on *Cornulaca monacantha*. On a permanent microscope slide in Euparal, deposited in SMNHTAU.

**Paratypes**: 1♀, 4♂, Nizzana 5 km N., Rt. 10, 30.9294, 34.3789, 12.iv.2002, N. Dorchin; 3♀, 2♂, Nahal Lavan, 30.9552, 34.3826, 7.v.1998, N. Dorchin; 2♀, Sede Halamish, 30.9219, 34.4053, 3.v.2020, A. Dorchin.

**Other material examined**: 2♂, Nizzana 5 km N., Rt. 10, 12.iv.2002, N. Dorchin; 2 larvae, Sede Halamish, 30.9219, 34.4053, 27.iii.2020, N. Dorchin; 4♀, 1♂, Sede Halamish, 30.9219, 34.4053, 3.v.2020, A. Dorchin.

**Distribution**: The galls of this species are found in great numbers in the single small area in the western Negev Desert where the host plant occurs in Israel. The galls were depicted in Houard [48], where they were reported from Tunisia and attributed to an unidentified gall midge. No doubt this species occurs also in Egypt.

**Etymology**: The species name refers to its spiny artichoke-shaped galls.

**Comments**: This species is distinct among the Israeli species for the narrow, almost-parallel-sided lobes of the male hypoproct and the completely fused male cerci. It is also notable that the frontoclypeal membrane bears only two long setae compared to numerous setae on each side in other Israeli species.

#### 3.2.9. *Asiodiplosis* *mohicana* Dorchin, New Species

**Host plants**: *Agathophora allopecuroides* (Delile) Fenzl ex Bunge.

**Gall and biology**: The large galls of this species are 1–1.5 cm in diameter and are often found in small groups. They are composed of fleshy cylindrical leaves that bear a long apical bristle typical to normal leaves of the host plant, and are mixed with long, white hairs that grow densely in the gall (Figure 24C,D). Some of these leaves are wider at their bases and are divided into several tapered lobes apically. Each gall contains 1–3 larval chambers at its base, composed of a somewhat rigid capsule surrounded by a group of thin, tapered scales. The galls are uncommon and found sporadically, and adults are rather difficult to rear. Except for one adult reared in mid-October, all adults were reared in February–April, which is the main activity season for this species.

**Adult description**: *Head*: Antennal flagellomeres 11–12 in both sexes. Male flagellomeres with short necks between nodes, occasionally without necks at all, and short distal necks about same length along antenna; circumfilar loops about half length of node. Female flagellomeres about same length along antenna, with slight constriction or without constriction medially, short necks to flagellomere 9 or 10, without neck thereafter; when 11 flagellomeres, apical flagellomere usually twice as long as preceding, with short apical projection. Frontoclypeal membrane with 5–6 setae on each side in male, 2–4 setae on each side in female. Palpus 1.5–2.5 times as long as wide.

*Thorax*: Wing length: 2.14–3.00 mm in female (*n* = 10), 2.28–2.90 mm in male (*n* = 6).

*Female abdomen*: Ovipositor 8.4–13.9 times as long as tergite 8 (*n* = 10). Setae on segment 9 0.5–1 as long as segment height.

*Male abdomen*: Terminalia: Cerci separated along distal half, with 3 apical setae on each side. Hypoproct lobes divided longitudinally into wide dorsal section and narrower ventral section with 3 long setae apically. Aedeagus tapered apically.

**Larva**: Not studied.

**Pupa** (Figure 23D,E): Antennal bases without any projections. Dorsal side of head case conspicuously inflated to form elongate, cylindrical lobe extending posteriorly.

**Holotype**: ♀, Israel, upper Nahal Ye’elim, 31.2389, 35.2358, 19.iv.1995, N. Dorchin, ex bud gall on *Agathophora allopecuroides*. On permanent microscope slide in Euparal, deposited in SMNHTAU.

**Paratypes**: 3♂, upper Nahal Ye’elim, 31.2389, 35.2358, 19.iv.1995, N. Dorchin; 1♂, Midreshet Ben-Gurion, upper Nahal Qarqash, 30.8538, 34.7694, 13.x.1997, N. Dorchin; 5♀, 1♂, 2 exuviae, HaMeshar, 30.4553, 34.9356, 27.ii.2020, N. Dorchin.

**Other material examined**: 1♂, upper Nahal Ye’elim, 31.2389, 35.2358, 19.iv.1995, N. Dorchin; 5♀, HaMeshar, 30.4553, 34.9356, 27.ii.2020, N. Dorchin.

**Distribution**: Uncommon species, found sporadically at several sites in the Negev desert.

**Etymology**: This species is named after the long, peculiar bulge on the pupal head, which is reminiscent of a Mohawk hairstyle.

**Comments**: The adults of this species resemble those of *A. largifica* but differ from them in the female antennal flagellomeres, which virtually lack medial constriction and in the male cerci, which are fused almost entirely. The pupae stand out among all other Israeli species for the conspicuous posterior extension of the head case, the nature of characters can be seen clearly in both pupae and exuviae.

#### 3.2.10. *Halodiplosis fugax* Dorchin, New Species

**Host plants**: *Anabasis articulata*.

**Gall and biology**: This species was reared only once from galls of *Asiodiplosis admirabilis* collected in early June. The immature stages went unnoticed when dissecting the galls, but a single larva was found actively crawling around on an empty gall. This larva was markedly different from larvae of the gall inducer, and was able to spring around using its well-developed spatula, as do larvae of many cecidomyiid species that leave their galls to pupate in the soil [3]. This observation suggests that larvae of this species develop as inquilines in *A. admirabilis* galls and leave them to pupate in the soil, as do other *Halodiplosis* species [46]. No other details about the life history of this species are known.

**Figure 25:**
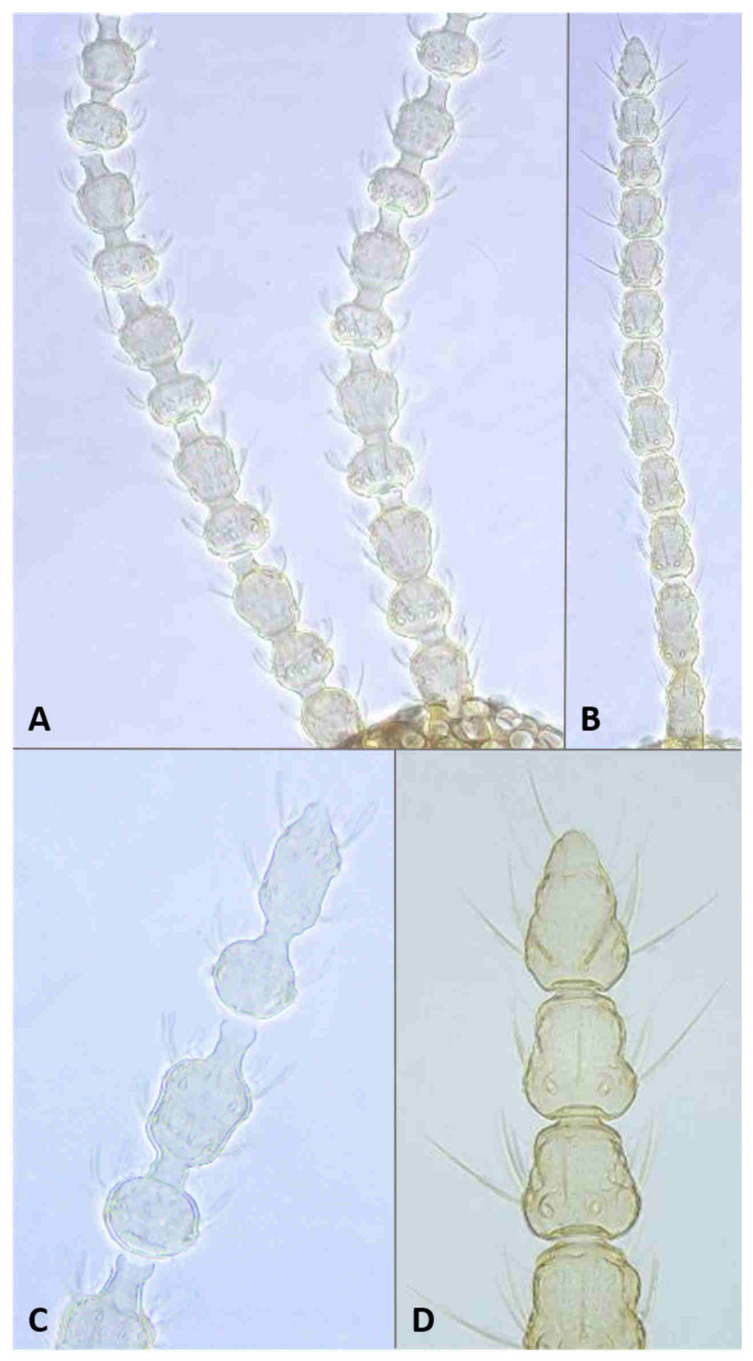
*Halodiplosis fugax*. (**A**). Male antennae; (**B**). Female antenna; (**C**). Male apical flagellomeres; (**D**). Female apical flagellomeres.

**Figure 26:**
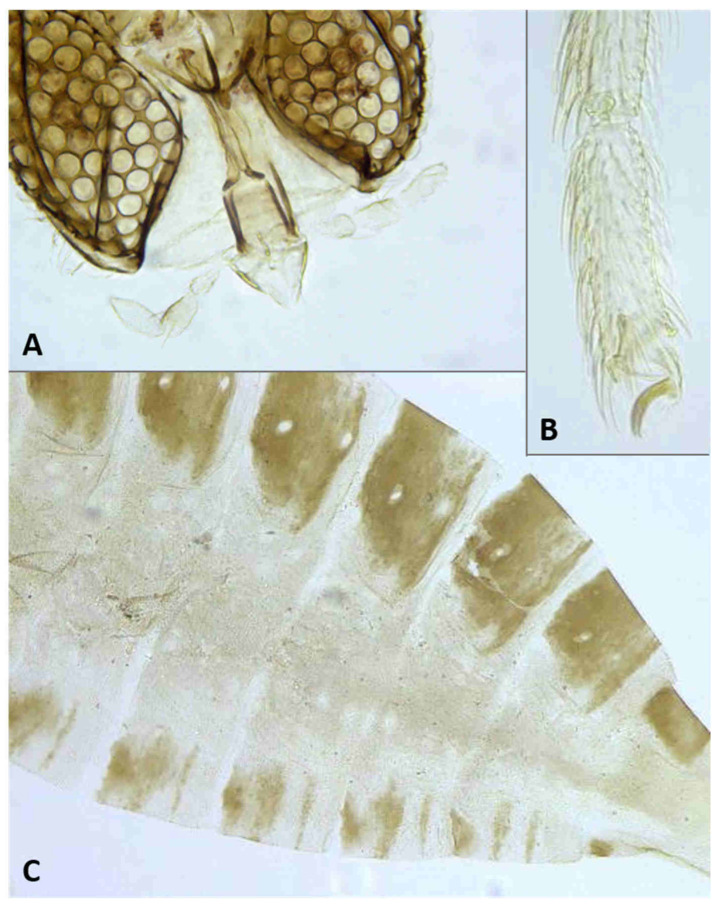
*Halodiplosis fugax*. (**A**). Head, dorsal; (**B**). Acropod, lateral; (**C**). Female abdomen, lateral.

**Adult description**: *Head*: Eye facets round. Antennal flagellomeres 12 in both sexes. Male flagellomere 1 effeminate, without neck between nodes, with short apical neck; flagellomere 2 with clearer separation between nodes; flagellomeres 3–11 (Figure 25A) composed of oval proximal node, wider than long, and cylindrical distal node, longer than wide; proximal nodes with one circumfilar whorl subtended by whorl of strong setae; distal nodes with two circumfilar whorls, separated by whorl of strong setae; circumfilar whorls about same length as proximal node; flagellomere necks about same length along antenna; flagellomere 12 tapered apically (Figure 25C). Female flagellomeres (Figure 25B) cylindrical, with clear medial constriction, first 5 consecutively shorter, remaining about same length, all with very short necks; each with proximal whorl of strong setae (grouped rather than lined on flagellomere 1) subtending closely appressed circumfilar whorl and distal whorl of strong setae; flagellomere 12 tapered apically (Figure 25D). Frontoclypeal membrane with 3–4 long setae on each side. Palpus (Figure 26A) 3-segmented, segment 1 about 1.5 times as long as wide, segments 2 and 3 about twice as long as wide, all setulose and bearing several long setae.

*Thorax*: Wing venation and setation as described above for *A. largifica*. Wing length: 1.75 mm in female (*n* = 1), 1.35 mm in male (*n* = 1). Claw untoothed, evenly curved, empodium and pulvilli rudimentary (Figure 26B).

*Female abdomen* (Figure 26C): Tergites 1–7 with anterior pair of sensory setae and posterior row of long setae; tergite 8 with pair of sensory setae the only vestiture. Sternites 2–7 pigmented along proximal half and posterior row of setae; sternite 8 with small pigmented patch and two setae. Ovipositor 11.8 times as long as tergite 8 (*n* = 1); segment 9 with numerous setae, less than half length of segment height; cerci closely appressed, with several setae 2–3 times as long as setae on segment 9.

**Figure 27:**
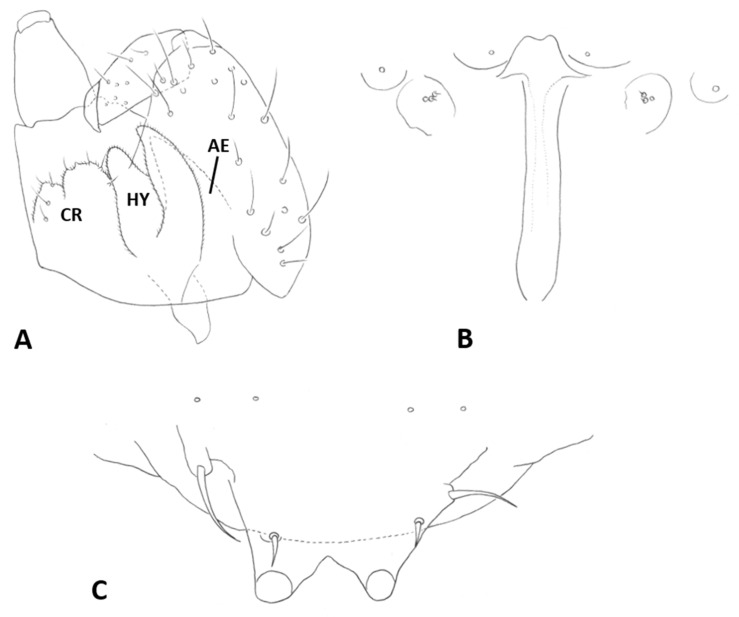
*Asiodiplosis fugax*. (**A**). Male terminalia, lateral; (**B**). Larval spatula and associated papillae; (**C**). Larval terminal abdominal segment. AE = aedeagus, CE = cerci, HY = hypoproct.

*Male abdomen*: Tergites 1–7 with anterior pair of sensory setae and posterior row of long setae; tergite 8 with small pigmented patch, pair of sensory setae, and single seta posteriorly. Sternites pigmented on proximal half, without anterior pair of sensory setae, with posterior row of setae and several setae medially; sternite 8 not differentiated from surrounding membrane. Terminalia (Figure 27A): Gonocoxite rectangular at base, narrows gradually toward apex, with numerous evenly distributed long setae. Gonostylus cylindrical, only slightly narrowed toward apex, evenly setose and setulose; apical tooth wide, brush-like, appears composed of closely packed setae. Cerci densely setulose, separated along apical half to form two short, rounded lobes, each with 4–5 long apical setae. Hypoproct completely separated into two cylindrical lobes, narrowed toward rounded apex, completely setulose, without strong setae apically. Aedeagus wide at base, narrowed toward rounded apex.

**Larva** (third instar): Elongate and bright orange. Integument covered by prominent triangular spicules. Spatula long-shafted, with trapezoidal, shallowly indented anterior lobe (Figure 27B). Sternal papillae asetose; 4 lateral papillae on each side of spatula, arranged in group of three papillae, two of which setiform, and one asetose papilla farther away. Ventral papillae minute, asetose, dorsal and pleural papillae with conspicuously long setae. Terminal segment (Figure 27C) with pair of massive, coniform pigmented papillae and two pairs of much-smaller setiform papillae.

**Pupa**: Unknown.

**Holotype**: ♂, Israel, Mamshit, 31.0342, 35.0674, 9.vi.1997, N. Dorchin, ex bud gall on *Anabasis articulata*. On permanent microscope slide in Euparal, deposited in SMNHTAU.

**Paratypes**: 1♀, 1 larva, Mamshit, 31.0342, 35.0674, 9.vi.1997, N. Dorchin.

**Distribution**: Reared only once from Mamshit in the Negev desert.

**Etymology**: “Fugax” is Latin for elusive or shy, with reference to the rarity of this species and its elusive life history.

**Comments**: Three of the 13 described species retained here in *Halodiplosis* develop as inquilines in galls on *Anabasis* host plants in Kazakhstan or Turkmenistan. These are *H. anabasidis* from galls of an unknown species on *A. aphylla* L., *H. constricta* (Mamaeva) from galls of *Asiodplosis iliensis* Marikovskij on *A. aphylla*, and *H. filipievi* (Fedotova) in galls of *Asiodiplosis anabasidis* (Fedotova) on *A. salsa* (Ledeb.) Benth. ex Volkens [47]. These species are generally similar to *H. fugax* morphologically, but it is unlikely that they are conspecific given the geographic distance and the different host plants. Morphological differences among these species include the uniquely pigmented female cerci of *H. constricta* that appear to be sclerotized, and the male hypoproct of *H. anabasidis* and *H. filipjevi* that is not deeply divided into two lobes as that of *H. fugax*. The male of *H. salsolae*, reared from galls on *Salsola tetragona* in Tunisia, was described as having nearly glabrous gonostyli [13], in contrast to the completely setulose gonostyli of *H. fugax*. The remaining species of *Halodiplosis*, all from Kazakhstan, do not develop in distinct galls and have other morphological and/or life-history attributes that distinguish them from *H. fugax* [46,47].

#### 3.2.11. Molecular Analysis

The results of the phylogenomic analysis based on the concatenated 16S and COI datasets are shown in Figure 18. The ML and BI analyses produced identical tree topologies; thus, Figure 18 presents the ML tree with both posterior probabilities and bootstrap-support values for each node. Our results clearly demonstrate the monophyly of the nine Israeli species of *Asiodiplosis*, with maximal support (i.e., PP = 1 and BS = 100%). Support values for deeper nodes were gradually more ambiguous as expected, but the ancestral position of *A. largifica* relative to all other species was strongly supported.

Intraspecific divergence within the nine *Asiodiplosis* species was low and never exceeded 1% in both mean K2P and uncorrected divergence (Table 3). Interspecific divergence, on the other hand, was an order of magnitude higher, and ranged between 10% and 20% in K2P values and 9% to 18% in the uncorrected values.

## 4. Discussion and Conclusions

Prior to this study, the genus *Halodiplosis* included 99 species, all but two of which from chenopod host plants [2]. Our exhaustive review of the relevant literature and work on the Israeli species revealed that this genus includes several entities that merit separate generic status. Following the reinstatement of *Onodiplosis* and *Desertomyia*, the remaining species in *Halodiplosis* could be divided into a large group of species (84) that form complex bud galls and a much smaller group of species (13) that develop in plant tissues without gall formation or are inquilines in galls of other species. These life-history attributes are consistently correlated with certain morphological characters, supporting the separation of these two groups into distinct genera. Because the type species of *Halodiplosis* belongs to the second group, this separation means that *Halodiplosis* is now limited to 14 species, including *H. fugax* described in the present study. The genus *Asiodiplosis*, reinstated here for all the bud gallers, currently includes 93 species, including the 9 described in the present work (Appendix A). Important morphological characters to be studied when describing new species in this genus include the shape of antennal flagellomeres in both sexes, setation of the ovipositor, shape of the male cerci and hypoproct, and attributes of the pupae and the larvae, which are currently available only for the Israeli species.

All *Asiodiplosis* species develop in complex bud galls, and the overwhelming majority were reported from a single host plant, attesting to a high level of host specificity. The occurrence in Israel of nine *Asiodiplosis* species suggests that related species can co-occur even within a small geographic area and even on the same host plant (*A. pillosaeconspicua* and *A. stellata*). Apparently, diversification in this genus occurred in response to the ample opportunities offered by chenopods in arid habitats, both in terms of species richness and abundance. Good examples found in Kazakhstan include *Haloxylon ammodendron* that hosts 14 *Asiodiplosis* species and *Anabasis salsa* with 13 species [2]. The results of our phylogenetic analysis suggest that species associated with the same plant genus are not necessarily closely related; thus, for example, the three species from *Anabasis* host-plants in Israel are more closely related to species from other host plants than they are to each other (Figure 27). More insight into the relationships and evolutionary trends in *Asiodiplosis* necessitates genetic data for the Central-Asian species, which are currently unavailable.

Although *Asiodiplosis* galls are generally similar in structure, species in this genus exhibit diverse life histories in terms of voltinism and abundance. Most species from Central Asia were reported to complete one generation per year with overwintering larvae; although many of them were sampled only once, so it is unclear how this was determined. Univoltine species are either rare (e.g., *A. anabasidicola* [47], *A. aristiformis* (Fedotova [15]), *A. dzhanokmenae* (Fedotova) [52] and *A. stellata*), or common (e.g., *A. hammadae* (Fedotova) [10], *A. raphidophitonis* (Fedotova) [8], and *A. mucronata*). Others have two generations per year, with galls that differ in morphology between the two generations. Examples include *A. mutabilis* Marikovskij from Kazakhstan [13] and *A. bimoda* from Israel. At the other end of the scale are abundant species that complete multiple generations per year, including *A. aphylla* (Fedotova) [53] and *A. nanophytonis* (Fedotova) [9] from Kazakhstan and *A. largifica* and *A. paradoxa* from Israel.

The genus *Halodiplosis*, as delimited here, includes species developing either as inquilines in *Asiodiplosis* galls or independently without apparent gall formation, leaving the plant to pupate in the soil [18,45,46,54]. When inquilinous in *Asiodiplosis* galls, the gall inducer and inquiline should be readily distinguishable from each other given the notable morphological differences between them. *Asiodiplosis* larvae are stout and sluggish, whereas *Halodiplosis* larvae (judging from those of *H. fugax*) are elongate and active. The adults can be readily distinguishable from each other by the three-segmented palpus, trifilar male flagellomeres, and large female cerci in *Halodiplosis*, compared to the one-segmented palpus, bifilar male flagellomeres, and very small female cerci in *Asiodiplosis*. Because no genetic data have been available for *Halodiplosis*, it is currently unknown if these two genera are closely related. It is also unclear why *Halodiplosis* is associated with certain galls or host plants but absent from others, as the known associations do not form a clear pattern. *Halodiplosis* species were found on host plants of different chenopod tribes and life forms, and while some are rare (e.g., *H. petrosimoniae* (Fedotova), *H. constricta*, *H. fugax*) [17,45], others were reported as common (e.g., *H. filipjevi*, *H. foliosae* (Fedotova) and *H. iljiniae* (Fedotova)) [46]. Clearly, more targeted sampling and research are needed in order to elucidate the life history of species in this genus and its systematic relations.

## Figures and Tables

**Table 1:** Samples used in the molecular analyses with collecting data and Genbank accession numbers. All samples except the outgroup (*Contarinia loti*) were collected in Israel.

Sample Name	Host Plant	Collection Data	Genbank Accession Numbers	
			COI	16S
*A. admirabilis* (10-1)	*Anabasis articulata*	Mezad Tamar 31.0275, 35.2417	OK330047	
*A. admirabilis* (10-2)	*Anabasis articulata*	Mezad Tamar 31.0275, 35.2417	OK330048	
*A. admirabilis* (11-1)	*Anabasis articulata*	Nahal Ashosh, Rt. 20 30.5114, 35.1800	OK330049	
*A. admirabilis* (11-2)	*Anabasis articulata*	Nahal Ashosh, Rt. 20 30.5114, 35.1800	OK330050	OK338929
*A. admirabilis* (17-1)	*Anabasis articulata*	Nahal Shezaf 30.7394, 35.2619	OK330063	OK338934
*A. admirabilis* (17-2)	*Anabasis articulata*	Nahal Shezaf 30.7394, 35.2619	OK330064	OK338935
*A. admirabilis* (36)	*Anabasis articulata*	Nahal Baraq, Rt. 90 30.4242, 35.1492	OK330089	
*A. bimoda* (2-1)	*Anabasis syriaca*	Arad 31.2731, 35.2300	OK330032	OK338917
*A. bimoda* (2-2)	*Anabasis syriaca*	Arad 31.2731, 35.2300	OK330033	OK338918
*A. bimoda* (4-2)	*Anabasis syriaca*	Zomet haNegev 31.0664, 34.8381	OK330036	OK338921
*A. bimoda* (5-1)	*Anabasis syriaca*	Nahal Ye’elim, Rt. 31 31.2389, 35.2358	OK330037	OK338922
*A. bimoda* (5-2)	*Anabasis syriaca*	Nahal Ye’elim, Rt. 31 31.2389, 35.2358	OK330038	OK338923
*A. bimoda* (7-1)	*Anabasis syriaca*	Nahal Zin, Rt. 40 30.7092, 34.7839	OK330041	OK338925
*A. bimoda* (7-2)	*Anabasis syriaca*	Nahal Zin, Rt. 40 30.7092, 34.7839	OK330042	
*A. bimoda* (19-1)	*Anabasis syriaca*	Nahal Zin, Rt. 40 30.7092, 34.7839	OK330065	OK338936
*A. bimoda* (19-2)	*Anabasis syriaca*	Nahal Zin, Rt. 40 30.7092, 34.7839	OK330066	OK338937
*A. bimoda* (20-1)	*Anabasis syriaca*	Avedat, Rt. 40 30.7931, 34.7675	OK330067	OK338938
*A. bimoda* (20-2)	*Anabasis syriaca*	Avedat, Rt. 40 30.7931, 34.7675	OK330068	OK338939
*A. bimoda* (32-1)	*Anabasis syriaca*	Nahal Zin, Rt. 40 30.7092, 34.7839	OK330083	OK338953
*A. bimoda* (32-2)	*Anabasis syriaca*	Nahal Zin, Rt. 40 30.7092, 34.7839	OK330084	OK338954
*A. bimoda*	*Anabasis syriaca*	Nahal Zin, Rt. 40 30.7092, 34.7839	MN191370	MN201487
*A. delicatula* (13-1)	*Haloxylon persicum*	En Yahav 30.6450, 35.2086	OK330052	OK338930
*A. delicatula* (13-2)	*Haloxylon persicum*	En Yahav 30.6450, 35.2086	OK330053	OK338931
*A. delicatula* (13-3)	*Haloxylon persicum*	En Yahav 30.6450, 35.2086	OK330054	OK338932
*A. delicatula* (31-1)	*Haloxylon persicum*	En Yahav 30.6450, 35.2086	OK330080	OK338950
*A. delicatula* (31-2)	*Haloxylon persicum*	En Yahav 30.6450, 35.2086	OK330081	OK338951
*A. delicatula* (31-3)	*Haloxylon persicum*	En Yahav 30.6450, 35.2086	OK330082	OK338952
*A. delicatula* (37)	*Haloxylon persicum*	Lotan 29.9811, 35.0856	OK330090	OK338955
*A. delicatula* (38)	*Haloxylon persicum*	Holot Kasuy 29.9839, 34.9786	OK330091	OK338956
*A. largifica* (14-1)	*Caroxylon vermiculatum*	Zomet Mezada, Rt. 90 31.3131, 35.3833	OK330055	
*A. largifica* (14-2)	*Caroxylon vermiculatum*	Zomet Mezada, Rt. 90 31.3131, 35.3833	OK330056	
*A. largifica* (14-3)	*Caroxylon vermiculatum*	Zomet Mezada, Rt. 90 31.3131, 35.3833	OK330057	
*A. largifica* (15-1)	*Caroxylon vermiculatum*	Nahal Parsa, Rt. 90 31.2126, 35.3571	OK330058	
*A. largifica* (15-2)	*Caroxylon vermiculatum*	Nahal Parsa, Rt. 90 31.2126, 35.3571	OK330059	
*A. largifica* (15-3)	*Caroxylon vermiculatum*	Nahal Parsa, Rt. 90 31.2126, 35.3571	OK330060	
*A. largifica* (22-1)	*Caroxylon vermiculatum*	HaMeshar, Rt. 40 30.4553, 34.9356	OK330070	
*A. largifica* (22-2)	*Caroxylon vermiculatum*	HaMeshar, Rt. 40 30.4553, 34.9356	OK330071	
*A. largifica* (34-1)	*Caroxylon vermiculatum*	Tomer, Rt. 90 32.0214, 35.4469	OK330085	
*A. largifica* (34-2)	*Caroxylon vermiculatum*	Tomer, Rt. 90 32.0214, 35.4469	OK330086	
*A. largifica* (35-1)	*Caroxylon incanescens*	Auja et-Tahta, Rt. 90 31.9278, 35.4678	OK330087	
*A. largifica* (35-2)	*Caroxylon incanescens*	Auja et-Tahta, Rt. 90 31.9278, 35.4678	OK330088	
*A. mohicana* (16-1)	*Agathophora allopecuroides*	Nahal Ye’elim, Rt. 31 31.2389, 35.2358	OK330061	OK338933
*A. mohicana* (16-2)	*Agathophora allopecuroides*	Nahal Ye’elim, Rt. 31 31.2389, 35.2358	OK330062	
*A. mohicana* (27-1)	*Agathophora allopecuroides*	HaMeshar, Rt. 40 30.4553, 34.9356	OK330077	OK338946
*A. mohicana* (27-2)	*Agathophora allopecuroides*	HaMeshar, Rt. 40 30.4553, 34.9356	OK330078	OK338947
*A. mucronata* (21-1)	*Cornulaca monacantha*	Nizzana, 5 km N, Rt. 10 30.9294, 34.3789	OK330069	OK338940
*A. mucronata* (28-1)	*Cornulaca monacantha*	Sede Halamish 30.9219, 34.4053	OK330079	OK338948
*A. mucronata* (28-2)	*Cornulaca monacantha*	Sede Halamish 30.9219, 34.4053		OK338949
*A. paradoxa* (1-1)	*Anabasis setifera*	Arad, 10 km E, Rt. 31 31.2547, 35.1697	OK330030	
*A. paradoxa* (1-2)	*Anabasis setifera*	Arad, 10 km E, Rt. 31 31.2547, 35.1697	OK330031	OK338916
*A. paradoxa* (3-1)	*Anabasis setifera*	Avenat, Rt. 90 31.6797, 35.4406	OK330034	OK338919
*A. paradoxa* (3-2)	*Anabasis setifera*	Avenat, Rt. 90 31.6797, 35.4406	OK330035	OK338920
*A. paradoxa* (6-1)	*Anabasis setifera*	Mezad Tamar 31.0275, 35.2417	OK330039	OK338924
*A. paradoxa* (6-2)	*Anabasis setifera*	Mezad Tamar 31.0275, 35.2417	OK330040	
*A. paradoxa* (8-1)	*Anabasis setifera*	Neot haKikkar 30.9519, 35.3636	OK330043	OK338926
*A. paradoxa* (8-2)	*Anabasis setifera*	Neot haKikkar 30.9519, 35.3636	OK330044	OK338927
*A. paradoxa* (9-1)	*Anabasis setifera*	Mezad Tamar 31.0275, 35.2417	OK330045	OK338928
*A. paradoxa* (9-2)	*Anabasis setifera*	Mezad Tamar 31.0275, 35.2417	OK330046	
*A. paradoxa*	*Anabasis setifera*	Enot Zuqim 31.7156, 35.4514	MN191298	MN201486
*A. pillosaeconspicua* (23-1)	*Caroxylon tetrandrum*	HaMeshar, Rt. 40 30.4553, 34.9356	OK330072	OK338941
*A. pillosaeconspicua* (23-2)	*Caroxylon tetrandrum*	HaMeshar, Rt. 40 30.4553, 34.9356	OK330073	OK338942
*A. pillosaeconspicua* (25-1)	*Caroxylon tetrandrum*	Nahal Qumeran, Rt. 90 31.7375, 35.4597	OK330074	OK338943
*A. pillosaeconspicua* (25-2)	*Caroxylon tetrandrum*	Nahal Qumeran, Rt. 90 31.7375, 35.4597	OK330075	OK338944
*A. stellata* (12-1)	*Caroxylon tetrandrum*	Nahal Zeruya, Rt. 90 31.4386, 35.3831	OK330051	
*A. stellata* (26)	*Caroxylon tetrandrum*	Nahal Zeruya, Rt. 90 31.4386, 35.3831	OK330076	OK338945
*Contarinia loti*	*Lotus corniculatus*	UK, Surrey, Ripley	MN191274	MN201465

**Table 2:** Comparative morphological characters of *Asiodiplosis* and *Halodiplosis* species in Israel. (Flag/s. = flagellommere/s).

Character	*Asiodiplosis* *largifica*	*Asiodiplosis* *paradoxa*	*Asiodiplosis* *admirabilis*	*Asiodiplosis bimoda*	*Asiodiplosis stellata*	*Asiodiplosis* *pillosaeconspicua*	*Asiodiplosis* *mohicana*	*Asiodiplosis mucronata*	*Asiodiplosis delicatula*	*Halodiplosis fugax*
Female wing length (mm)	1.50–2.26	1.28–2.37	1.60–2.50	1.75–2.69	1.58–3.10	1.26–2.15	2.13–3.01	2.57–3.02	2.54–2.94	1.75
Male wing length (mm)	1.76–2.69	1.45–2.35	1.62–2.65	2.12–2.86	2.62–3.66	1.70–2.45	2.28–2.90	2.51–3.02	2.76–3.04	1.35
Male flag. Number	11–12	12	12	12	12	12	11–12	12	12	12
Female flag. Number	11–12	11–12	10–11	11	11–12	11–12	11–12	11–12	11	12
Apical flags. Fused?	Yes	No	No	No	Yes	Yes	Yes	Yes	No	No
Male flag. Necks	Successively longer along proximal half	Successively longer along proximal half	Same length throughout antenna	Same length throughout antenna	Successively longer	Same length throughout antenna	Same length throughout antenna	Somewhat successively longer	Successively longer	Same length throughout antenna
Length of circumfilar loops	Half length of node	As long as node	Half length of node	Half length of node	As long as node	Half length of node	Half length of node	Half length of node	Half length of node	Half length of node
Female flag. constriction	In flags. 1–5	In flags. 1–3	In flags. 2–5	In flags. 2–7	In flags. 1–7	In flags. 1–5	Slight or none	In flags. 1–11	In flags. 2–7	In flags. 1–12
Female flag. necks	Flags 1–5	Yes	Yes	Yes	Flags 1–7	Yes	Yes	Yes	Flags 1–7	Not really
Apical flag. projection	No	No	Yes	Occasionally slight	No	No	Occasionally slight	Occasionally slight	Yes	No
Setae on frontoclypeal membrane (each side)	4–8 female 5–14 male	5–6	1–2	5–7	5–12 female 15–20 male	2–4 female 3–6 male	5–6	1	3–8	3–4
Ovipositor length	13.2–21.2	5.6–16.9	8.58–11.31	9.01–15.13	9.31–13.04	6.16–13.60	8.4–13.9	9.2–11.8	6.46–16.21	11.8
Ovipositor setae (segment 9)	0.3 as long as Oviopositor width	As long as Oviopositor width	0.5 as long as Ovipositor width	0.5–0.7 as long as ovipositor width	0.3 as long as ovipositor width	0.3 as long as ovipositor width	0.5–1 as long as ovipositor width	0.5–1 as long as ovipositor width	Much longer than ovipositor width	0.3 as long as ovipositor width
Male cerci, shape, and setation	Separate on distal half. 5–6 setae	Separate apically. 3 setae	Separate apically. 3–4 setae	Separate to base	Separate at very top	Separate at very top	Almost completely fused	Completely fused	Separate apically	Separate apically
Male hypoproct, shape and, setation (on each side)	Longitudinal division. Wide; 10 setae	Longitudinal division. Narrow; 3 setae	Longitudinal division. Wide; 3–4 setae	Longitudinal division. Wide; 3 setae	Slight division. Wide; many setae	Slight division. Wide; many setae	Longitudinal division. Wide; 3 setae	Longitudinal division. Narrow; parallel-sided 3–4 setae	Longitudinal division. Rectangualr; apical “teeth”	Separate to base; without apical setae
Spatula	Rudimentary	No	Unknown	Yes	Yes	Yes	Unknown	No	Yes	Yes
Pupal “horns”	No	Yes	No	No	Minute tips	Minute tips	No. posterior lobe	No	No	Unknown

**Table 3:** Estimates of average evolutionary divergence over sequence pairs within and between groups. Intra- and inter-specific distances within *Asiodiplosis* are based on the COI dataset involving the 62 de novo sequences generated in the current study with a total length of 564 positions. Mean p-distances between (above-diagonal) and within (diagonal and after-slash) groups as well as K2P distances between (below-diagonal) and within (diagonal and before-slash) groups are shown. Evolutionary analyses were conducted in MEGA7 [41]. Groups (species) correspond to the clades denoted in Figure 27.

Species	*A. paradoxa*	*A. bimoda*	*A. admirabilis*	*A. stellata*	*A. delicatula*	*A. largifica*	*A. mohicana*	*A. mucronata*	*A. pillosaeconspicua*
*A. paradoxa*	0.00/0.00	0.09	0.12	0.17	0.10	0.12	0.09	0.12	0.15
*A. bimoda*	0.10	0.01/0.01	0.13	0.18	0.12	0.14	0.10	0.13	0.15
*A. admirabilis*	0.13	0.14	0.00/0.00	0.17	0.14	0.15	0.14	0.15	0.15
*A. stellata*	0.20	0.20	0.20	0.00/0.00	0.16	0.15	0.17	0.16	0.18
*A. delicatula*	0.10	0.13	0.16	0.18	0.00/0.00	0.15	0.12	0.14	0.15
*A. largifica*	0.13	0.16	0.17	0.17	0.16	0.01/0.01	0.10	0.14	0.14
*A. mohicana*	0.10	0.11	0.15	0.19	0.13	0.11	0.00/0.00	0.11	0.13
*A. mucronata*	0.13	0.15	0.17	0.17	0.16	0.15	0.12	0.01/0.01	0.15
*A. pillosaeconspicua*	0.16	0.17	0.17	0.20	0.16	0.16	0.14	0.17	0.01/0.01

## Data Availability

Sequences were deposited in GenBank—respective accession numbers are provided in Table 1. Other data are available upon request from the corresponding author.

## Appendix A

An updated generic assignment and new combinations of Cecidomyiini species from Amaranthaceae and Fabaceae resulting from this study are presented below. Original species names are given in parentheses where relevant. The specific epithets of some species transferred by Gagné [20] to *Halodiplosis* from other genera were modified by him to avoid homonymy resulting from that act. These epithets are reverted here to their original form and are given in parentheses after the original name where relevant.

***Onodiplosis*** **Felt, 1916**

*O. sarcobati* Felt, 1916

***Desertomyia*** **Marikovskij, 1975**

*D. dissimmetrica* Marikovskij, 1975 (*Halodiplosis dissimmetrica* of Gagné 2004)

*D. caraganae* Fedotova, 1991 (*Contarinia caraganae* of Gagné 2004)

***Halodiplosis* Kieffer, 1912**

*H. anabasidis* (Fedotova, 1990) (as *Bojalodiplosis anabasidis*; *Halodiplosis anabasidia* of Gagné 2004)

*H. arbocarpis* (Fedotova, 1985) (as *Bojalodiplosis arbocarpis*)

*H. arthrophytumis* (Fedotova, 1993) (as *Bojalodiplosis arthrophytumis*; *H. arthrophytumidis* of Gagné 2004)

*H. capsica* (Fedotova, 1990) (as *Bojalodiplosis caspicus*)

*H. constricta* (Mamaeva, 1980) (as *Micrasiodiplosis constricta*)

*H. filipjevi* (Fedotova, 1993) (as *Halocnemomyia filipjevi*)

*H. foliosae* (Fedotova, 1993) (as *Halocnemomyia foliosae*)

*H. fugax* Dorchin

*H. halimocnemii* (Fedotova, 1993) (as *Halocnemomyia halimocnemii*)

*H. halogetonis* (Fedotova, 1992) (as *Bojalodiplosis halogetonis*)

*H. iljiniae* (Fedotova, 1993) (as *Halocnemomyia iljiniae*)

*H. petrosimoniae* (Fedotova, 1993) (as *Halocnemomyia petrosimoniae*)

*H. salsolae* Kieffer, 1912

*H. schnitnikovi* (Marikovskij, 1965) (as *Halocnemomyia schnitnikovi*)

***Asiodiplosis* Marikovskij, 1955**

(All but the species described in this study are listed under *Halodiplosis* in Gagné (20)).

*A. admirabilis* Dorchin

*A. aestivas* Marikovskij, 1953 (as *Asidiplosis aestivas*)

*A. anabasidicola* Fedotova 1989 **new combination** (as *Halodiplosis anabasidicola*)

*A. anabasidigemmae* Fedotova, 1989 **new combination** (as *Halodiplosis anabasidigemmae*)

*A. anabasidis* (Fedotova, 1989) **new combination** (as *Halodiplosis anabasidis*)

*A. aphylla* (Fedotova, 1992) **new combination** (as *Halodiplosis anabasidis*)

*A. araratica* (Mirumian, 1991) **new combination** (as *Halodiplosis araratica*)

*A. aristiformis* (Fedotova, 1993) **new combination** (as *Halodiplosis aristiformis*)

*A. arthrophytumis* (Fedotova, 1992) **new combination** (as *Halodiplosis arthrophytumis*)

*A. aurantiaca* (Fedotova, 1992) **new combination** (as *Halodiplosis aurantiaca*)

*A. bacilliformis* (Fedotova, 1991) **new combination** (as *Halodiplosis bacilliformis*)

*A. balchashensis* (Fedotova, 1993 **new combination** (as *Halodiplosis bachashensis*)

*A. bassiae* (Fedotova, 1993) **new combination** (as *Halodiplosis bassiae*)

*A. biennis* (Marikovskij, 1955) **new combination** (as *Haloxylophaga biennis*)

*A. bimoda* Dorchin

*A. botryoidea* (Fedotova, 1993) **new combination** (as *Halodiplosis botryoidea*)

*A. buzaczensis* (Fedotova, 1993) **new combination** (as *Halodiplosis buzazensis*)

*A. caspica* (Fedotova, 1993) **new combination** (as *Halodiplosis caspicus*; *H. caspicana* of Gagné 2004)

*A. ceratoidis* (Fedotova, 1994) **new combination** (as *Halodiplosis ceratoidis*)

*A. climacopterae* (Fedotova, 1993) **new combination** (as *Halodiplosis climacopterae*)

*A. conmixta* (Mamaev & Pak, 1990) **new combination** (as *Halodiplosis conmixta*)

*A. consociata* (Marikovskij, 1955) **new combination** (as *Haloxylophaga consociata*)

*A. czuensis* (Fedotova, 1993) **new combination** (as *Halodiplosis czuensis*)

*A. delicatula* Dorchin

*A. densipila* (Marikovskij, 1957) **new combination** (as *Tyloceramyia densipila*)

*A. deserta* Marikovskij, 1955

*A. dzhanokmenae* (Fedotova, 1993) **new combination** (as *Halodiplosis dzhanokmenae*)

*A. eremobia* (Mamaev & Pak, 1989) **new combination** (as *Halodiplosis eremobia*)

*A. fedtschenkovi* (Marikovskij, 1965) **new combination** (as *Haloxylophaga fedtschenkovi*)

*A. festinans* Marikovskij, 1955

*A. floripara* (Mamaev, 1972) **new combination** (as *Haloxylophaga floripara*)

*A. germinantis* (Fedotova, 1991) **new combination** (as *Halodiplosis germinantis*)

*A. hammadae* (Fedotova, 1993) **new combination** (as *Halodiplosis hammadae*)

*A. hebes* (Marikovskij, 1957) **new combination** (as *Monarthropselaphus hebes*)

*A. hirsuta* Mamaev, 1972

*A. hirta* Marikovskij, 1955

*A. hodukini* Marikovskij, 1965

*A. Iliensis* Marikovskij, 1955

*A. indurentis* (Fedotova, 1993) **new combination** (as *Halodiplosis indurentis*)

*A. infestans* (Marikovskij, 1955) **new combination** (as *Haloxylophaga infestans*)

*A. inflorescentiae* (Fedotova, 1990) **new combination** (as *Halodiplosis inflorescentiae*)

*A. inornata* (Marikovskij, 1955) **new combination** (as *Halxylophaga inornata*)

*A. insularis* (Fedotova, 1993) **new combination** (as *Halodiplosis insularis*)

*A. invisibilis* (Fedotova, 1989) **new combination** (as *Halodiplosis invisibilis*)

*A. largifica* Dorchin

*A. latentis* (Mamaev & Pak, 1989) **new combination** (as *Halodiplosis latentis*)

*A. ludmilae* (Fedotova, 1994) **new combination** (as *Halodiplosis ludmilae*)

*A. magna* (Mamaev, 1972) **new combination** (as *Haloxylophaga magna*)

*A. meridiana* Marikovskij, 1956

*A. mohicana* Dorchin

*A. morbosa* (Mamaev & Pak, 1989) **new combination** (as *Halodiplosis morbosa*)

*A. mucronata* Dorchin

*A. mutabilis* Marikovskij, 1955

*A. nanophytonigemmae* (Fedotova, 1990) **new combination** (as *Halodiplosis nanophytonigemmae*)

*A. nanophytonis* (Fedotova, 1990) **new combination** (as *Halodiplosis nanophytonis*)

*A. noxia* Marikovskij, 1955

*A. orientalis* (Fedotova, 1994) **new combination** (as *Halodiplosis orientalis*)

*A. oxyglumis* (Fedotova, 1993) **new combination** (as *Halodiplosis oxyglumis*)

*A. palpata* Marikovskij, 1955

*A. panderiae* (Fedotova, 1992) **new combination** (as *Halodiplosis panderiae*)

*A. paradoxa* Dorchin

*A. petrosimoinae* (Fedotova, 1994) **new combination** (as *Halodiplosis petrosimoniae*; *H. petrosimoinana* of Gagné 2004)

*A. pillosaeconspicua* Dorchin

*A. primoveris* Marikovskij, 1953

*A. propria* Marikovskij, 1955

*A. repetekensis* (Kaplin, 1990) **new combination** (as *Halodiplosis repetekensis*)

*A. rhaphidophytonis* (Fedotova, 1989) **new combination** (as *Halodiplosis rhaphidophytonis*)

*A. rosaria* Mamaev, 1972

*A. salsae* Marikovskij, 1955

*A. salsola* Marikovskij, 1955

*A. salsolicola* (Marikovskij, 1955) **new combination** (as *Haloxylophaga salsolicola*)

*A. slsuginea* Mamaev, 1972

*A. savojskella* Marikovskij, 1955

*A. saxauli* (Kaplin, 1991) **new combination** (as *Halodiplosis saxauli*)

*A. slyvkini* (Fedotova, 1992) **new combination** (as *Halodiplosis slyvkini*)

*A. sperandus* (Fedotova, 1989) **new combination** (as *Halodiplosis sperandus*)

*A. sphaerobia* Marikovskij, 1955

*A. stackelbergi* Marikovskij, 1955

*A. steinbergi* (Marikovskij, 1955) **new combination** (as *Tyloceramyia steinbergi*)

*A. stellata* Dorchin

*A. strobiliformis* (Fedotova, 1991) **new combination** (as *Halodiplosis strobiliformis*)

*A. sympegmae* (Fedotova, 1990) **new combination** (as *Halodiplosis sympegmae*)

*A. syrdarjensis* Marikovskij, 1955

*A. truncata* (Fedotova, 1992) **new combination** (as *Halodiplosis truncata*)

*A. truncatula* (Mamaev, 1972) **new combination** (as *Haloxylophaga truncatula*)

*A. tsherkesi* (Kaplin, 1990) **new combination** (as *Halodiplosis tsherkesi*)

*A. ulkulkalkani* Marikovskij, 1955

*A. unica* (Fedotova, 1992) **new combination** (as *Halodiplosis unicus*)

*A. urceolatus* (Fedotova, 1993) **new combination** (as *Halodiplosis urceolatus*)

*A. uzenensis* (Fedotova, 1993) **new combination** (as *Halodiplosis uzenensis*)

*A. vernalis* Marikovskij, 1955

*A. vicina* Marikovskij, 1955

*A. zajsanicus* (Fedotova, 1993) **new combination** (as *Halodiplosis zajsanicus*)
